# Salt Tolerance and Physiological Responses at the Seedling, Vegetative, and Reproductive Stages of Thai Jasmine Rice KDML105 and Its Genetically Improved Variety (RD73) and Line (TSKC1-144)

**DOI:** 10.3390/plants15172635

**Published:** 2026-08-28

**Authors:** Nuttida Khampookhiaw, Oracha Khianpho, Supranee Santanoo, Dechudom Pamuta, Piyada Theerakulpisut

**Affiliations:** 1 Salt-Tolerant Rice Research Group, Department of Biology, Faculty of Science, Khon Kaen University, Khon Kaen 40002, Thailand; nuttkh@kku.ac.th (N.K.); oracha.kp@gmail.com (O.K.); suprsa@kku.ac.th (S.S.); 2 Ubon Ratchathani Rice Research Center, Rice Department of Thailand, Mueang Ubon Ratchathani District, Ubon Ratchathani 34000, Thailand; dechudom.p@rice.mail.go.th

**Keywords:** flag-leaf physiology, photosynthesis, salt tolerance, *Saltol* QTL, sodium exclusion, yield

## Abstract

The level of salt tolerance of rice varies with the developmental stage. The information on the tolerance of rice at each stage is valuable for breeding and planning in cultural management to obtain the optimal growth and yield in salt-affected areas. The objectives of this study were to compare the salt tolerance and physiological- and yield-related responses at three growth stages of the salt-sensitive Thai jasmine rice, KDML105, with its genetically improved variety RD73 (a registered commercial variety) and TSKC1-144 (a breeding line), both containing Pokkali-derived salt-tolerant QTL. The young hydroponically grown seedlings of TSKC1-144 treated with 150 mM NaCl were highly tolerant, while KDML105 was highly sensitive and RD73 was moderately tolerant. At the vegetative stage, KDML105 exhibited more growth and leaf physiological damage, showing the highest percentage reductions in the net photosynthesis rate (Pn), leaf relative water content (RWC), and shoot and total plant dry weight, but the highest increase in leaf electrolyte leakage (EL) and the highest leaf Na^+^/K^+^ ratio. During the reproductive phase, salt stress did not significantly induce physiological damage to the flag leaves, except for Pn, which was significantly reduced, particularly for KDML105. Both RD73 and TSKC1-144 exhibited lower biomass reductions and higher yields and yield components than KDML105. Compared with TSKC1-144, RD73 produced a lower grain number panicle^−1^, grain weight panicle^−1^, and lower 100-grain weight but 34% more panicles; therefore, it yielded a higher grain weight plant^−1^ (27.57 cf. 20.93 g). The most prominent trait that conferred a greater salt tolerance to RD73 and TSKC1-144 compared with KDML105 was the more efficient Na^+^ exclusion. Taking their tolerance at all growth stages into consideration, RD73 and TSKC1-144 are deemed suitable for growing under rain-fed conditions where the intensity of the soil salinity fluctuates throughout the growing season.

## 1. Introduction

Climate change and global warming have caused an alarming rise in the frequency and severity of environmental challenges that severely threaten food security, including droughts, flooding, extreme high temperatures, and salinity. The Global Map of Salt-Affected Soils (GSASMap) estimates that more than 424 Mha of topsoil (0–30 cm) and 833 Mha of subsoils (30–100 cm) distributed across all continents are salt-affected [1]. Due to the impact of climate change, 14.73% of global soils have experienced a significant increase in salinity between 1980 and 2018, and long-term droughts have contributed to the development of saline soils in 6.78% of global dry regions [2]. Most areas with salinity-affected soils are located in Africa and Asia, especially in South and Southeast Asia [3]. In Thailand, salt-affected areas cover 1.904 Mha, of which about 1.841 Mha is found in the northeastern part, covering approximately 18% of the total land area [4]. Rice is the major export commodity of Thailand, and the northeast is the largest rice-producing area, accounting for 50% of the total rice cultivation land, but the rice yield is the lowest among all of the regions, partly due to the impact of soil salinity [5]. The rice yield has significantly decreased with the increase in soil salinity, but the yield has varied over the years. In a severely saline area in the northeast region, an extremely low yield (<100 g m^−2^) was observed in the dry year of 2018, while, in the wet years of 2017 and 2019, the yields were between 200 and 400 g m^−2^ [6]. In a farmer’s saline rice field in this region, the yield of KDML105, the most important rice variety for export and domestic consumption, was dramatically reduced by 58% compared with a nearby non-saline field [7], posing a threat to food security and community well-being.

Salt stress adversely affects susceptible plants by suppressing the growth of young leaves and accelerating the senescence of mature leaves, ultimately leading to plant death [8]. A high salt concentration lowers the soil’s water potential, imposing, primarily, osmotic stress, which prevents root water uptake and reduces cell turgor pressure, leading to stunted growth and wilting [9]. To alleviate osmotic stress, plants employ tolerance mechanisms such as the synthesis, transport, and accumulation of low-molecular-weight compatible osmolytes (e.g., proline and sugars) to maintain cellular turgor pressure [10]. Rice varieties with a greater salt tolerance are equipped with a higher ability to lower their cellular osmotic potential by accumulating compatible solutes, thereby retaining water absorption and maintaining cellular turgor [11]. Secondarily, high salinity levels result in ionic stress due to the toxicity of the accumulated sodium ions (Na^+^), which interferes with the uptake of essential nutrients like K^+^ and Ca^2+^, leading to the inhibition of enzyme activity, metabolic disturbance, and membrane integrity and functioning [12]. In rice, maintaining a relatively high K^+^/Na^+^ or low Na^+^/K^+^, especially in the shoots and leaves, is a key factor in developing salt tolerance [13]. Among the multitude of metabolic processes, photosynthesis is particularly sensitive to salt stress, directly affecting growth and grain yield. Salt stress impairs photosynthesis through an osmotic effect that causes stomatal closure, hence limiting CO_2_ absorption, as well as the direct effect of Na^+^ toxicity, which damages the photosynthetic machinery, particularly that of photosystem II [14]. Thirdly, the concerted declines in CO_2_ fixation and Calvin cycle activity, PSII photochemical efficiency, and electron transport lead to an overproduction of reactive oxygen species (ROS), resulting in oxidative stress [8,15]. Although ROS at appropriate levels function as signaling molecules regulating various physiological processes, excessive ROS accumulation is toxic and leads to cellular damage, growth inhibition, and a reduced grain yield and quality [16,17]. The development of salt tolerance in rice requires an increased capability of these core physiological mechanisms, including osmotic adjustment, the prevention of toxic Na^+^ accumulation, and the elimination of ROS. Each process involves the multi-layered complex regulation of stress signaling and the network of gene expression and functioning.

Enhancing salt tolerance and increasing the yield of rice when cultivated in saline soils through conventional and molecular breeding is one of the most powerful strategies for alleviating the problems of deteriorating rice production in many countries. In modern molecular breeding, one of the most successful approaches involved the introgression of the major *Saltol* QTL (derived from Pokkali landrace) and *SKC1* gene (derived from Nona Bokra landrace) into the genome of elite rice through marker-assisted breeding [18]. *Saltol* is a major QTL located on chromosome 1, identified from a recombinant inbred line (RIL) population obtained from a cross between Pokkali (the salt-tolerant indica landrace) and IR29 (the salt-sensitive cultivar) [19]. The *Saltol* QTL contained numerous transcription factors and structural genes which conferred a salt tolerance capacity to young rice seedlings by maintaining the low Na^+^/K^+^ ratio in the shoots. The key structural gene within *Saltol* is *SKC1*, later identified as *OsHKT1;5*, which encoded a plasma-membrane localized sodium-selective transporter, is predominantly expressed in xylem parenchyma tissues of roots and leaf sheaths. It plays a crucial role in rice salt tolerance by controlling Na^+^ transport, unloading Na^+^ from the xylem vessels, and preventing toxic accumulation in young leaves [20,21]. Many commercial salt-tolerant varieties of elite rice introgressed with *Saltol* through marker-assisted breeding have been released in several countries, for example, BR11-*Saltol* and BRRI dhan28-*Saltol* from Bangladesh; Pusa 44-NILs and Sarjoo 52-NILs from India; and Bacthom7-*Saltol* from Vietnam [22,23,24]. Khao Dawk Mali 105 (KDML105) is the world-renowned rice variety originating from Thailand, characterized by its distinctive aroma and excellent cooking qualities, but its production is severely limited by its salt sensitivity [25,26]. After several years of breeding efforts, the Rice Department of Thailand released RD73 in 2017 as the first registered salt-tolerant rice variety in KDML105 genetic background [25]. The RD73 variety was obtained by an introgression of *Saltol* from FL496 (a recombinant inbred line that inherited *Saltol* QTL from Pokkali) into thea KDML105 genome through marker-assisted backcross breeding (MABC). It was characterized by a high level of salt tolerance, a higher yield production than KDML105 in saline soils, and its similarity to KDML105 in agronomic traits and grain quality, and is recommended for growing in saline rice fields in the northeast Thailand [25,26]. Recently, a pyramided line, TSKC1-144, was developed through MABC by the introgression into the KDML105 genome both the drought-tolerant QTL on chromosome 8 (DT-QTL8 from drought-tolerant donor, DH103) and the salt-tolerant gene *SKC1* (from Pokkali). The TSKC1-144 line was originated from a cross between CSSL94 (an improved line of KDML105 containing DT-QTL8) as the recurrent parent and RGD4 (an improved line of KDML105 containing the *SKC1* gene from Pokkali) as the salt-tolerant gene donor. The resultant hybrid was backcrossed twice to CSSL94, and, from a set of BC_2_F_3_ population, TSKC1-144 was selected as being tolerant to both drought and salinity at the seedling stage [27]. In a saline field in northeast Thailand, RD73 and TSKC1-144 exhibited a 45% and 32% reduction in grain weight plant^−1^ compared with that in the nearby non-saline field while KDML105 suffered a 58% reduction [7]. However, the salt tolerance at other developmental stages and the physiological responses have not been evaluated among these rice varieties. The soil salinity fluctuates dynamically across the entire growing season in rain-fed rice-growing areas of northeast Thailand, and rice may encounter salt stress at any developmental stage. Therefore, the evaluation of the salt tolerance across all growth stages is more consistent with local production conditions, and is valuable for practical application in the breeding and cultural management of rice growing in saline fields.

Although improved salt-tolerant varieties with the KDML105 genetic background are available, i.e., RD73 (the *Saltol* QTL-introgression variety) and TSKC1-144 (the *SKC1*-DT QTL8 pyramided line), there is still a lack of systematic comparative research on these materials across three key growth stages. Therefore, the objectives of this study were primarily to evaluate the salt tolerance at the seedling stage of KDML105, RD73, and TSKC1-144. Secondarily, the physiological and growth responses to salt stress of these varieties were evaluated at the vegetative stage. Finally, during the reproductive phase, the physiology of flag leaves at different stages together with the biomass and yield in greenhouse-grown rice subjected to salt stress were evaluated. Differential salt responses at different growth stages will provide a guideline for the utilization of rice varieties to suit the changing saline environments through the growing seasons. Moreover, the data from this study will provide an important guideline for further genetic improvement in TSKC1-144.

## 2. Results

### 2.1. Salt Tolerance at the Seedling Stage

**Figure 1:**
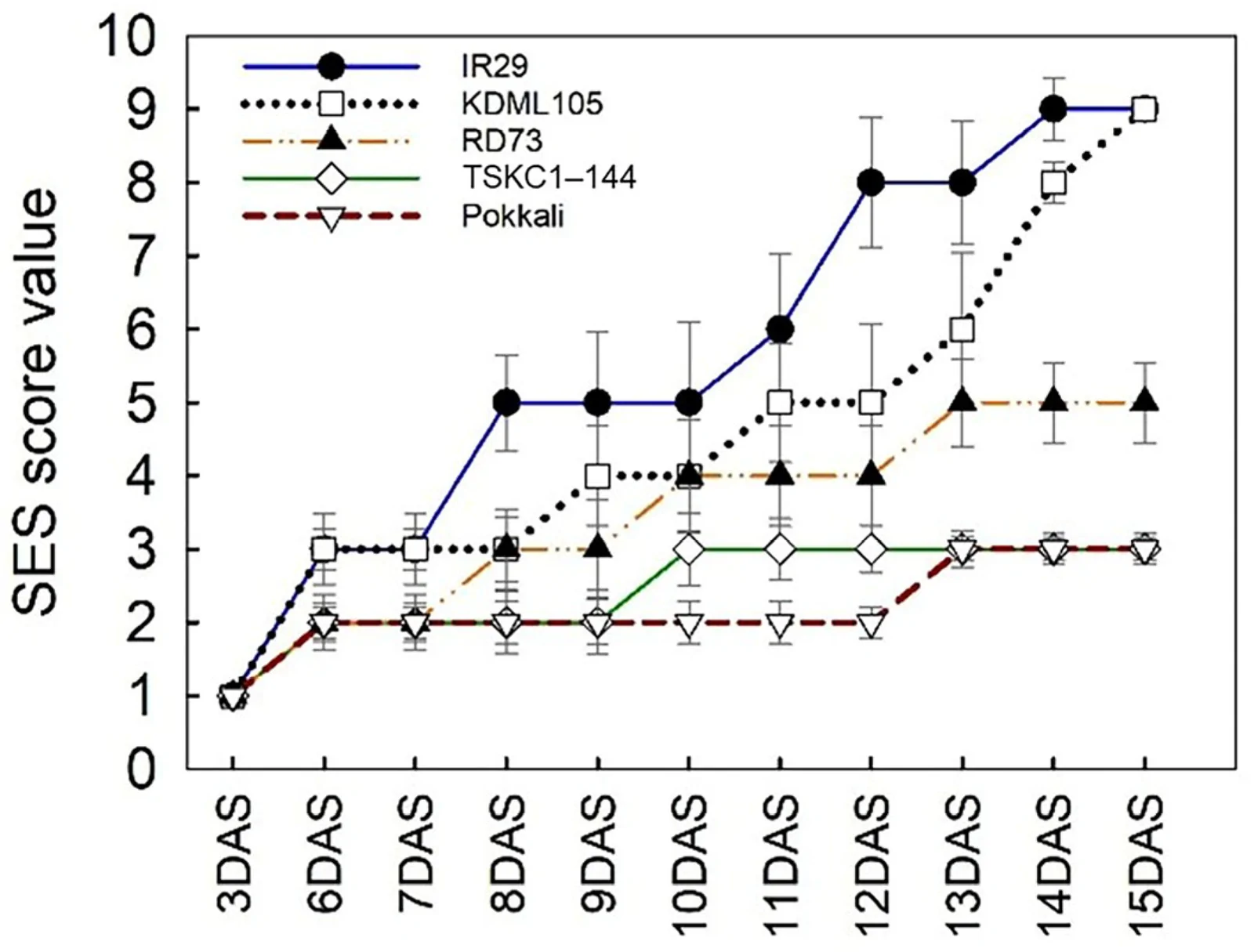
Changes in SES scores indicating salt injury of young rice seedlings after being treated with 150 mM NaCl in hydroponic solutions for 15 days (DAS, days after salt exposure). Data are the mean ± SE (*n* = 4).

**Figure 2:**
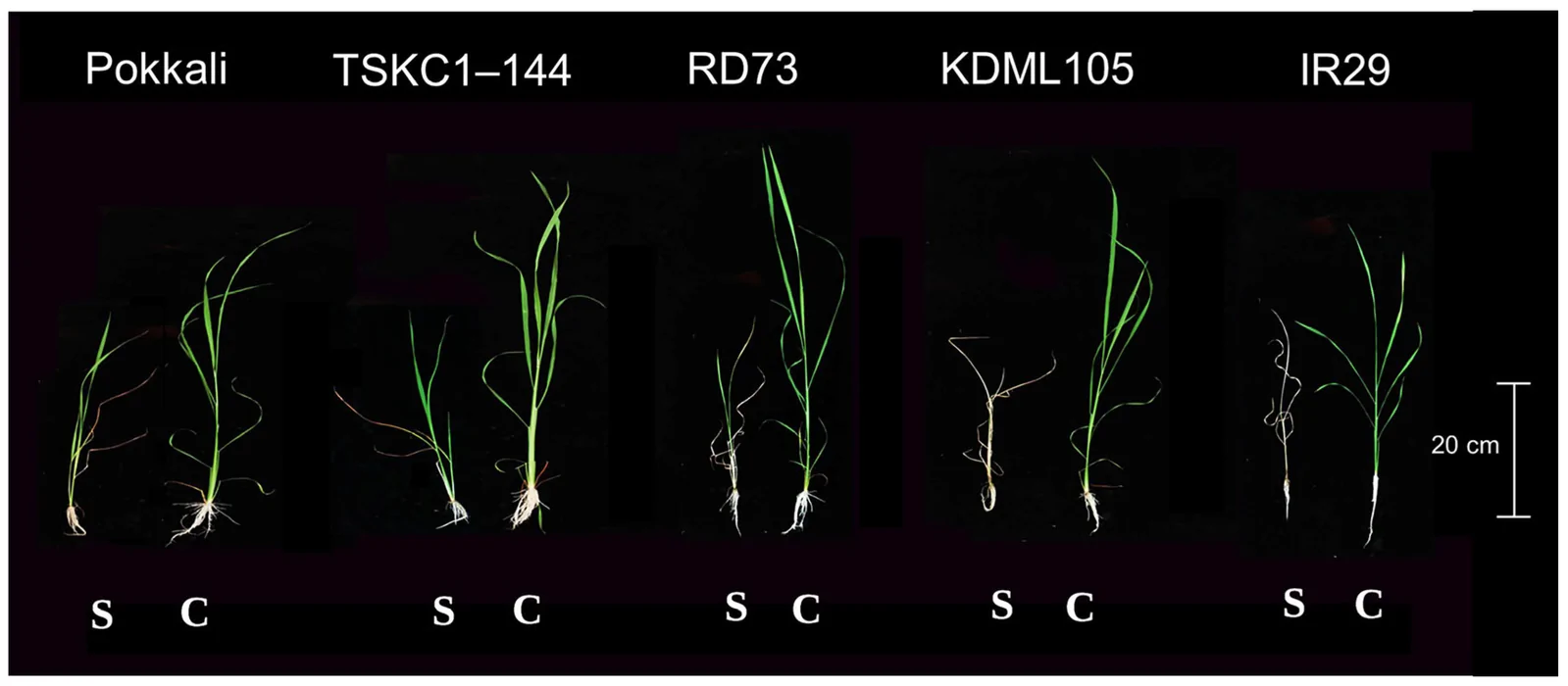
Morphology of rice seedlings 15 days after being treated with salt stress in the hydroponic cultures (S, salt stressed seedlings; C, control seedlings).

After three days of the NaCl introduction, all the rice varieties/lines were still healthy with green leaves and showed no signs of injury, achieving an SES score of 1 based on the standard evaluation system (SES) scores established by the International Rice Research Institute (IRRI, 2021) (Figure 1). On day 6, the lower leaves of IR29 and KDML105 started to wilt at the tips while other varieties/lines appeared normal. On day 9, while Pokkali, TSKC1-144, and RD73 still showed no signs of injury with the mean SES scores between 1.7 and 2.25, IR29 and KDML105 recorded higher scores of 4.3 and 3.7, respectively, with more old leaves dying and some younger leaves stopping their elongating. On day 12, IR29 recorded the mean SES score of 7.25 with almost all leaves dead and only the youngest leaf remaining green, while KDML105 retained more green leaves than IR29, showing the mean SES score of 4.65. On day 12, Pokkali and TSKC1-144 were still healthy with most leaves remaining green, more old leaves of RD73 had died and fewer leaves remained green. On day 15, almost all plants of IR29 and KDML105 died, showing the mean SES score of 8.85 and 8.55, respectively (Figure 1 and Figure 2). In contrast, seedlings of Pokkali and TSKC1-144 were alive with three to four leaves remaining green. Compared with TSKC1-144, RD73 was more damaged than TSKC1-144, having more dead leaves and a lower number of green leaves. Therefore, at the young seedling stage, KDML105 showed a similar level of salt sensitivity as IR29 (highly sensitive), TSKC1-144 achieved a similar tolerance level as Pokkali (highly tolerant), and RD73 was moderately tolerant.

### 2.2. Salt Tolerance and Physiology at the Vegetative Stage

The treatment with 150 mM NaCl acted as a severe salinity stress on rice at the vegetative stage, leading to profound disruption in many physiological aspects with substantial variations in tolerance levels of the sensitive KDML105 and the genetically improved variety, RD73, and line TSKC1-144.

#### 2.2.1. Photosynthesis, Water Relation, and Carbohydrate Contents in Leaves

After nine days of exposure to NaCl stress, the three rice varieties/lines showed no significant differences in chlorophyll content between the salt-stressed and the control groups (Table 1). In contrast, carbon assimilation was severely retarded, resulting in a 62% reduction in the net photosynthesis rate (Pn) of all the varieties/lines after only three days of salt exposure. After six days of stress, the Pn of KDML105 continued declining at the 65% reduction level compared with the controls, while RD73 and TSKC1-144 slightly recovered, exhibiting a slightly higher Pn (from 7.25 to 8.76, and from 7.31 to 8.98 µmol CO_2_ m^−2^ s^−1^, for RD73 and TSKC1-144, respectively). On day 9 after stress, the Pn was reduced by 49, 34, and 44% from the controls in KDML105, RD73, and TSKC1-144, respectively. An analysis of the non-structural carbohydrate in rice leaves revealed that the sugar contents in stressed leaves were statistically similar to those of the controls with KDML105, showing a higher level of increase than the others. In contrast, the starch content in KDML105 and RD73 leaves significantly increased by 23% and 19%, respectively, while TSKC1–144 also showed a slight increase of 4% (Table 1).

The presence of salts in the soils lowered the soil water potential, leading to the lower ability of plants to absorb water, leading to a significant reduction in the leaf relative water content (RWC) of KDML105 (9% reduction) and RD73 (7% reduction). TSKC1-144 was more efficient in maintaining the leaf water status, showing a non-significant reduction in RWC. Among the varieties, KDML105 showed the most severe membrane damage, with a 67% increase in leaf electrolyte leakage (EL), while EL of RD73 and TSKC1-144 remained similar to the control plants.

#### 2.2.2. Sodium and Potassium Ions in Leaves and Roots

The sodium ion (Na^+^) content and ratio between sodium and potassium ions (Na^+^/K^+^) in leaf tissues are the key determinants of the ability of plants to exclude the toxic sodium ions from the metabolically active leaf cells and to maintain ion homeostasis. All three varieties/lines grown in the control condition had similar a Na^+^, K^+^, and Na^+^/K^+^ in the leaves, although TSKC1-144 tended to have a noticeably lower Na^+^ and Na^+^/K^+^ (Table 2). Under salt stress, all the varieties/lines accumulated Na^+^ to a significantly higher content than the controls. Among the varieties/line, TSKC1-144 had a significantly lower Na^+^ and higher K^+^, resulting in a significantly lower leaf Na^+^/K^+^ than RD73 and KDML105, which had similar levels for all three ion parameters. In roots, salt-stressed TSKC1-144 had the highest Na^+^ (1.74%), followed by KDML105 (1.61%), and RD73 (1.45%), while the K^+^ contents were similar but the root Na^+^/K^+^ was significantly different among varieties/lines. Opposite to that in the leaf, TSKC1-144 had the highest Na^+^/K^+^ (2.76) in the root which was significantly higher than that of KDML105 (2.23) and RD73 (1.90).

#### 2.2.3. Growth and Biomass

An exposure of NaCl for 9 days posed strong inhibitory effects on rice growth, resulting in significant reductions in all growth and biomass parameters (Table 3). In the non-stress condition, all three rice varieties/lines had a similar root length and plant height. Under stress, root lengths were reduced by 36–49% as compared with the controls, but all the varieties/lines still had a similar length, while plant heights were 15–24% reduced and significantly different among varieties/lines, with the most inhibitory effects in RD73. The shoot fresh weight was significantly reduced in all the varieties/lines but the shoot dry biomass was significantly reduced only in KDML105 (49% reduction). However, the shoot dry biomass of all the varieties/lines were not significantly different, although TSKC1-144 had the highest biomass (2.15 g), followed by RD73 (1.85 g) and KDML105 (1.45 g). Both the root fresh and dry weight were most reduced in RD73 (75%); however, the dry root biomass of all the varieties/lines under stress were not significantly different. The total plant dry biomass was most reduced in KDML105 (52% reduced), while that of RD73 and TSKC1-144 were reduced by 34–35%. However, the total biomass of all the varieties/line was not significantly different, varying from 1.80 g in KDML105 to 2.40 g in TSKC1-144.

### 2.3. Physiology of Flag Leaves and Yield at the Reproductive Stage

Some physiological aspects of rice flag leaves were observed to change with the advancement in developmental stages as well as the effects of salt stress. In addition, different varieties/lines responded differently at different ages, leading to a variation in grain yield.

#### 2.3.1. Chlorophyll and Photosynthesis Performance of Flag Leaves

**Figure 3:**
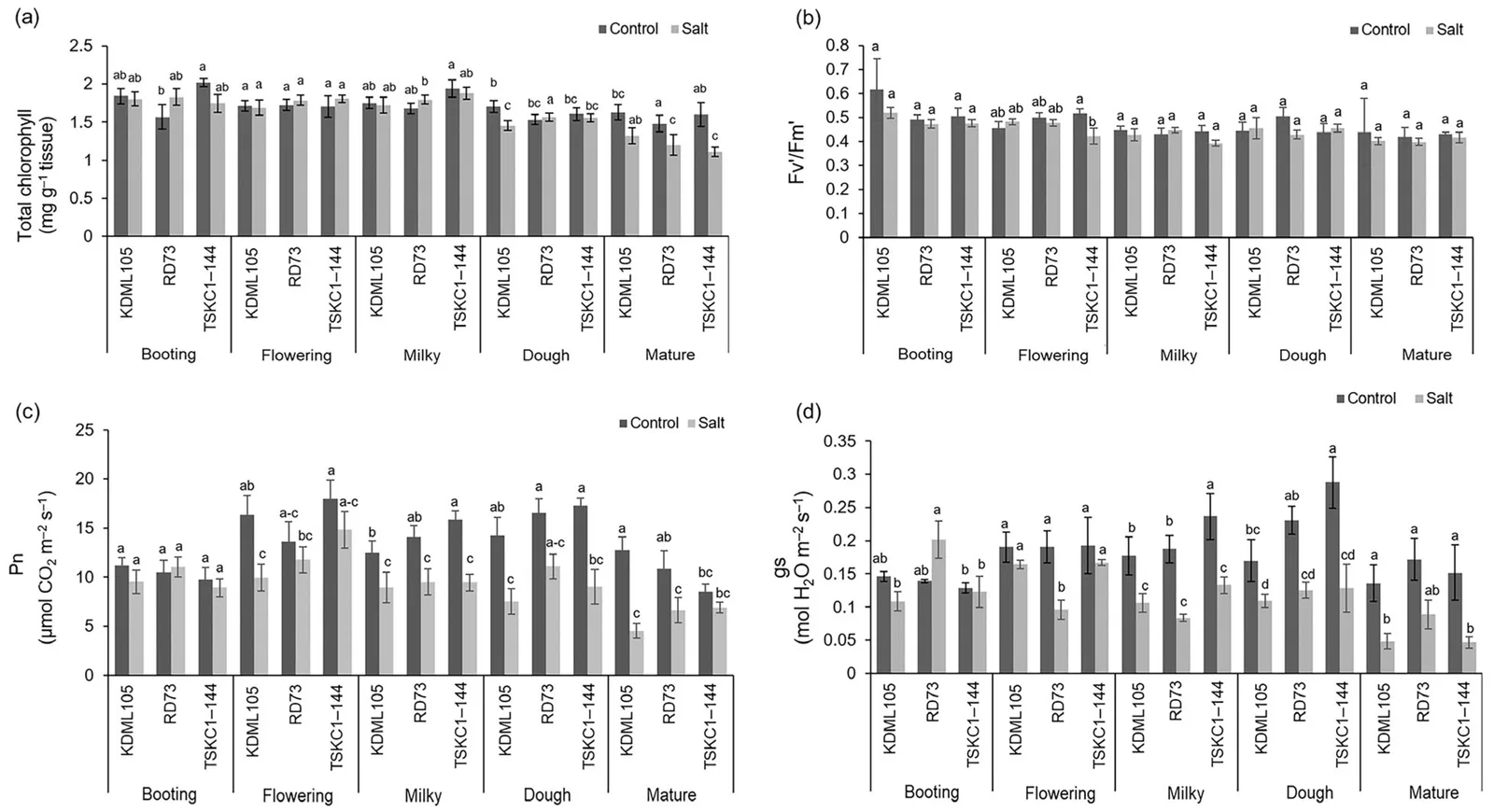
Total chlorophyll contents (**a**), maximum quantum yield of PS II in the light (Fv′/Fm′) (**b**), net photosynthesis rate (Pn) (**c**), and stomatal conductance (gs) (**d**) of the flag leaves of rice at different developmental stages under the control and salt stress conditions. At each growth stage, significant differences (*p* ≤ 0.05) among varieties/lines and growth conditions are indicated by different lowercase letters. Data are the mean ± SE (*n* = 4).

The total chlorophyll content in flag leaves of the control plants remained stable from booting to the milky stage, but then slightly reduced at the dough and mature stage. At the dough stage, salt stress caused a significant reduction (19%) in the chlorophyll content of KDML105, but, contrastingly, a significant increase (31%) in RD73. At maturity, the chlorophyll content of salt-stressed RD73 and TSKC1-144 significantly reduced by 33 and 31%, respectively (Figure 3a; Table S1). The maximum quantum yield of PSII in the light (Fv′/Fm′) which reflects the maximum efficiency of the photochemical energy conversion of PSII in the light-adapted state remained stable in the control plants from booting to the mature stage, indicating the stability of the structural and functional integrity of the PSII of flag leaves even at maturity. At each growth stage, salt stress caused a slight non-significant reduction in the Fv′/Fm′ values. However, the Fv′/Fm′ values of the salt-stressed plants tended to significantly decline with age, from an average of 0.489 at booting to 0.405 at maturity (Figure 3b; Table S1). This indicated that the light reaction of flag leaves, including the photochemical efficiency of PSII, was not negatively affected by salt stress throughout the development of flowers and grains.

As shown in Figure 3c and Table S1, the net photosynthesis rate (Pn) of the control plants varied, with age being the lowest in the young plant at booting and the older plant at maturity (averaged at 10.48 and 10.67 µmol CO_2_ m^−2^ s^−1^, respectively). During the flowering and dough stage, the average Pn rates were 15.97 and 16.01 µmol CO_2_ m^−2^ s^−1^, which was approximately 30% higher than that at the booting and mature stage. Salt stress generally caused a reduction in the Pn rates of all the three varieties/line at all developmental stages. On average, the Pn of salt-stressed plants was the highest at the flowering stage (12.18 µmol CO_2_ m^−2^ s^−1^) and the lowest at maturity (6.02 µmol CO_2_ m^−2^ s^−1^). At the milky stage, while the chlorophyll content and the photochemical efficiency of PSII were not significantly affected by salt stress (Figure 3a,b), the CO_2_ fixation was significantly reduced in all the three varieties/lines (Figure 3c), i.e., a 28, 33, and 40% reduction in KDML105, RD73, and TSKC1-144, respectively. Among the varieties/line, the Pn of KDML105 was the most impacted by salt stress, showing a significant reduction at every stage, from flowering (39%), milky (28%), dough (47%), and maturity (64%). However, a significant reduction in RD73 occurred only at the milky (33%) and maturity (39%) stages, and that in TSKC1-144 at the milky (40%) and dough (48%) stages (Figure 3c; Table S1). The reduction in Pn was primarily the result of stomatal closure as indicated by the consistently large decrease in stomatal conductance (gs) during flowering to maturity (Figure 3d). However, the intercellular CO_2_ concentration (Ci) under stress in most cases did not differ significantly from those in the control condition (Table S1). Therefore, metabolic functions in the chloroplast may also be affected, leading to the inefficiency of the CO_2_ uptake into the chloroplast for fixation.

#### 2.3.2. Relative Water Content (RWC) and Electrolyte Leakage (EL) of Flag Leaves

**Figure 4:**
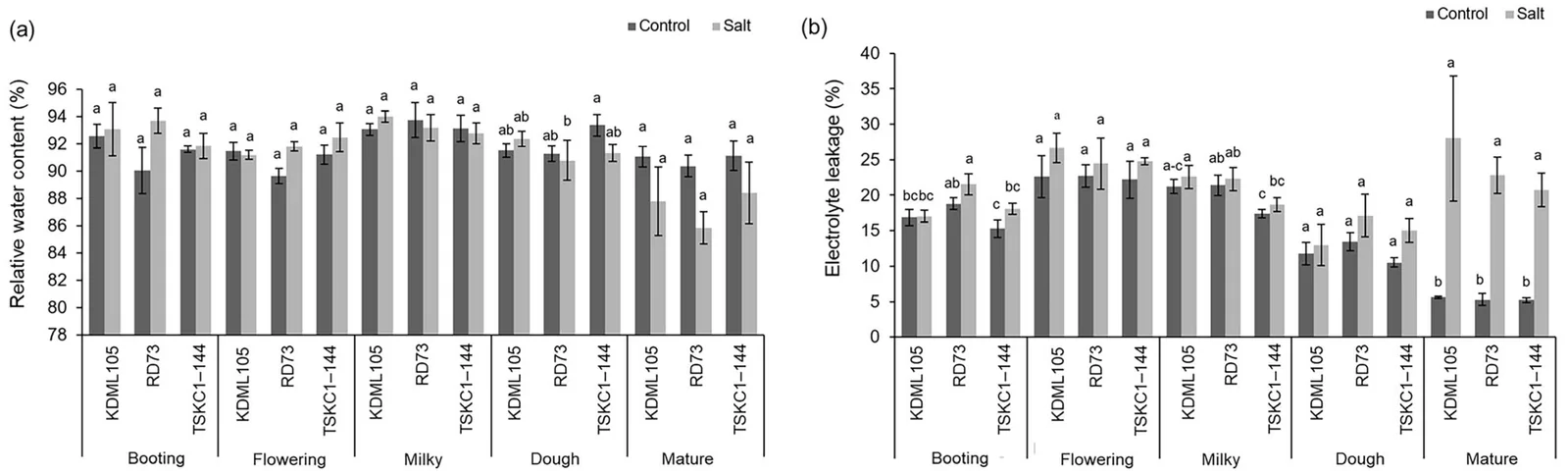
Relative water content RWC (**a**) and electrolyte leakage (El) (**b**) of the flag leaves of rice at different developmental stages under control and salt stress conditions. At each growth stage, significant differences (*p* ≤ 0.05) among varieties/lines and growth conditions are indicated by different lowercase letters. Data are the mean ± SE (*n* = 4).

A high concentration of NaCl causes a reduction in soil water potential, induces water stress at plant roots, and restricts plant water uptake, leading to cell dehydration and membrane damage. In this study, flag leaves of the salt-stressed plants of all the varieties/lines were able to efficiently maintain the water status between 91.47 and 93.30% from booting to the dough stage, and, later, it was significantly reduced to 87.34% only at maturity (Figure 4a; Table S2). The electrolyte leakage (EL), which indicated the integrity of the cellular membrane, showed an average of a 6–12% non-significant increase due to salt stress during booting to the milky stage, a 25% increase at the dough stage, and a maximum of a 343% increase at maturity (Figure 4b; Table S2). These results indicated that flag leaves of rice efficiently maintained their water status throughout the reproductive phase with no significant impact of salt stress, while the cell membrane integrity was damaged by salinity only at maturity.

#### 2.3.3. Effects of Salt Stress on H_2_O_2_, Thiobarbituric Acid Reactive Substances (TBARSs), and Proline in Flag Leaves

**Figure 5:**
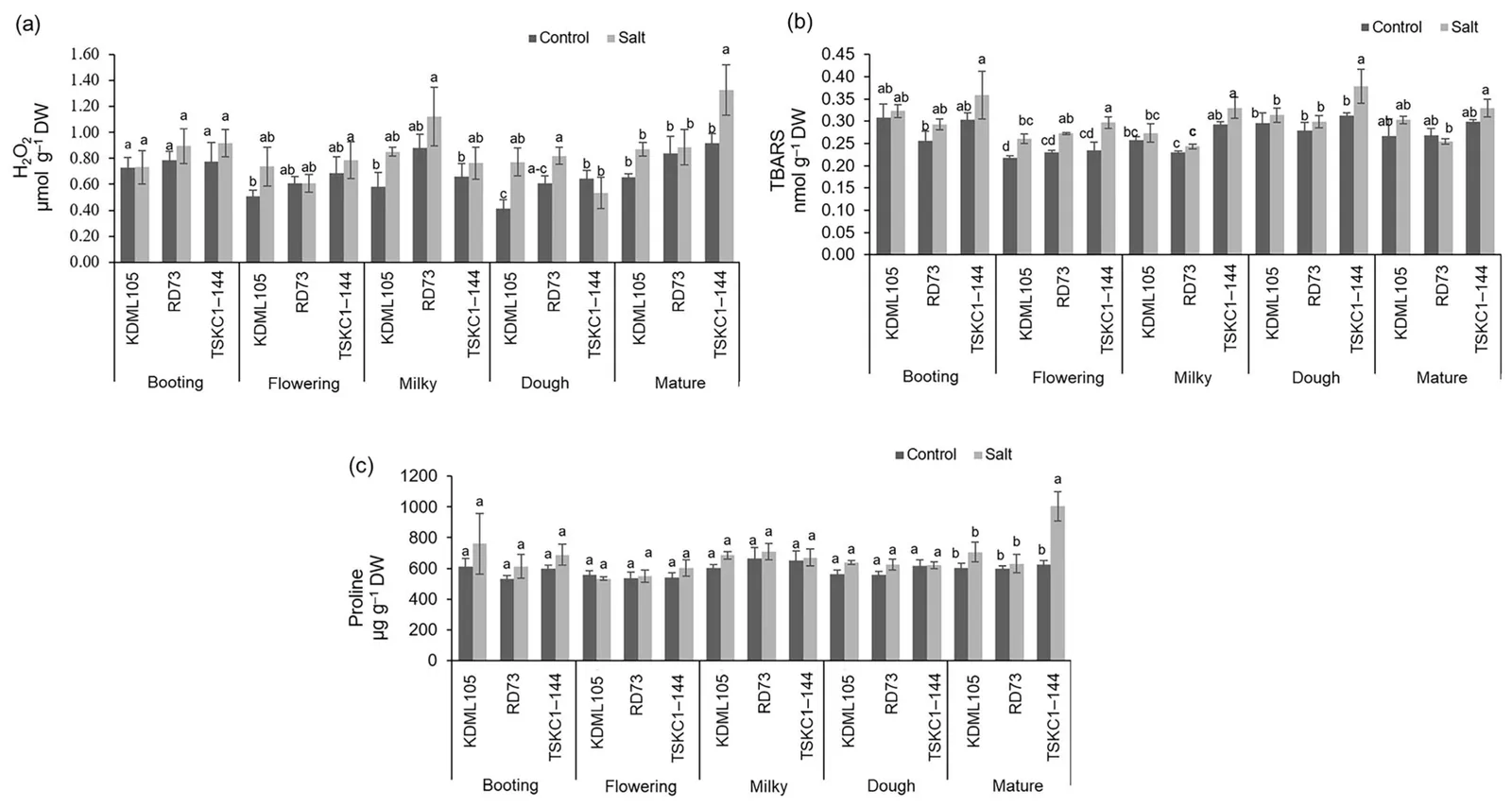
Hydrogen peroxide (H_2_O_2_) (**a**), thiobarbituric acid reactive substances (TBARS) (**b**), and proline (**c**) of the flag leaves of rice at different developmental stages under control and salt stress condition. At each growth stage, significant differences (*p* ≤ 0.05) among varieties/lines and growth conditions are indicated by different lowercase letters. Data are the mean ± SE (*n* = 4).

Salt stress leads to an accumulation of reactive oxygen species (ROS), including H_2_O_2_, which actively oxidize cellular lipids, resulting in a lipid peroxidation chain reaction, releasing malondialdehyde (MDA) as one of the byproducts which is detected by the TBARS assay. In this study, the H_2_O_2_ contents in the flag leaves of salt-stressed rice were mostly higher than those of the controls, although not significantly different in most cases (Figure 5a; Table S3). Moreover, H_2_O_2_, on average, tended to increase with age, being the highest at maturity in both the control (0.80 µmol g^−1^ DW) and stressed set (1.03 µmol g^−1^ DW). The difference among varieties/lines was evident at maturity, where TSKC1-144 produced significantly higher H_2_O_2_ than the others. Similarly, the concentrations of TBARS in the flag leaves of salt-stressed plants were consistently higher than those in the control plants, although with significant differences only at the flowering stage for all three varieties/lines (Figure 5b; Table S3). Proline, which generally accumulated in rice under drought and salt stress, also showed a trend of enhanced accumulation upon salt stress (Figure 5c; Table S3). However, only at maturity, the proline content in salt-stressed TSKC1-144 was significantly higher than the control. This indicated that salt stress induced a slight production of ROS such as H_2_O_2_, but not to a level so high that will cause significant damage to the flag leaves.

#### 2.3.4. Sugar and Starch Content in Flag Leaves

**Figure 6:**
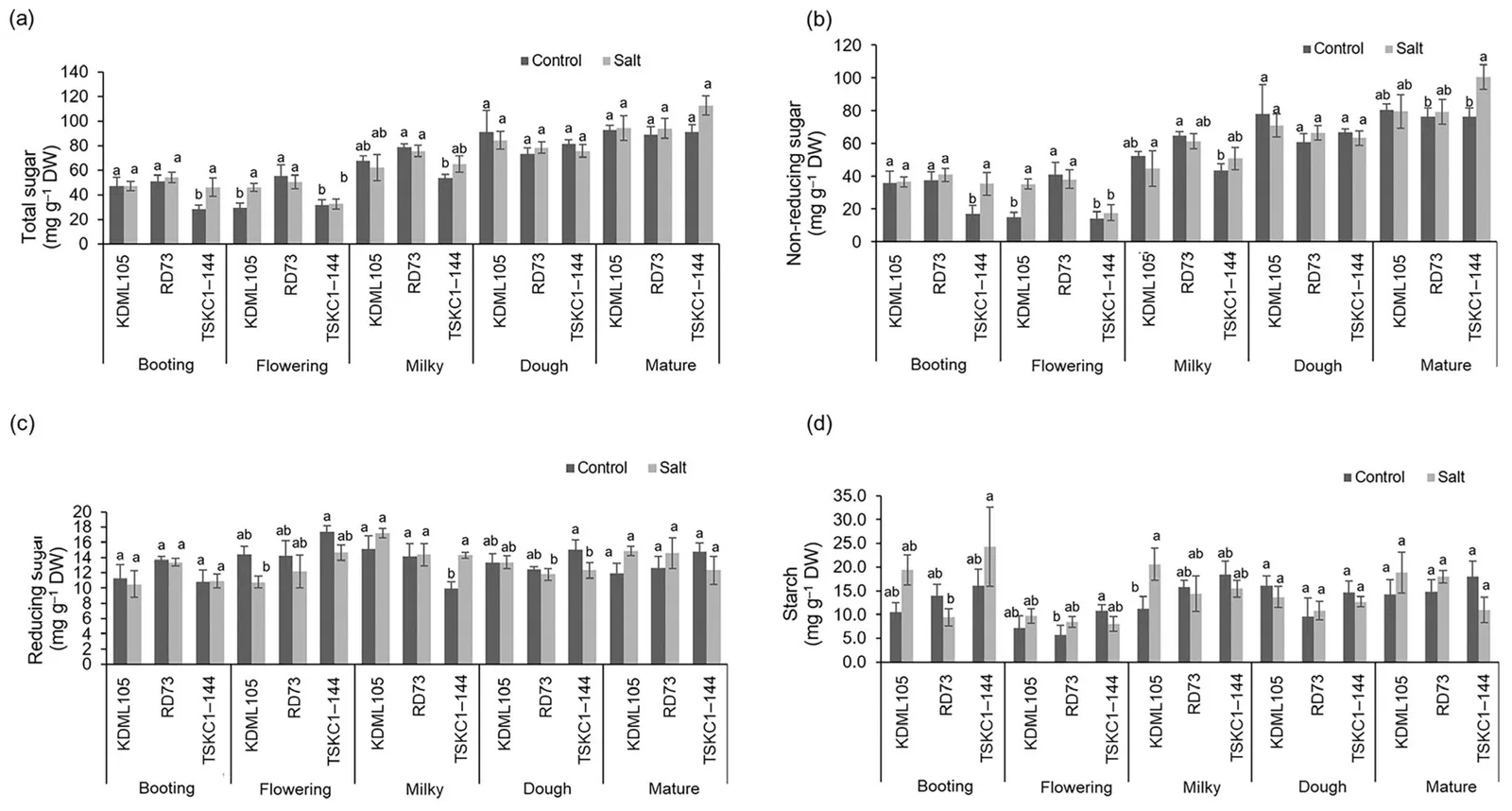
Total sugar (**a**), non-reducing sugar (nRS) (**b**), reducing sugar (RS) (**c**), and starch content (**d**) of flag leaves of rice at different developmental stages under control and salt stress conditions. At each growth stage, significant differences (*p* ≤ 0.05) among varieties/line and growth conditions are indicated by different lowercase letters. Data are the mean ± SE (*n* = 4).

The mean of the total sugar in flag leaves across three varieties/lines continuously increased with age, from booting (42.27 and 49.28 mg g^−1^ DW for control and stressed plants, respectively) to maturity (90.97 and 100.39 mg g^−1^ DW for control and stressed plants, respectively) (Figure 6a; Table S4). Similar patterns of changes were evident in the case of the non-reducing sugar (Figure 6b; Table S4). The effects of the salt treatment on the sugar contents varied with the varieties and developmental stage, mostly with non-significant differences between the control and stressed plants. At the booting stage, the mean starch content across varieties/lines was 13.54 mg g^−1^ DW for the control and 17.73 mg g^−1^ DW for the stressed plants (Figure 6d; Table S4). At the flowering stage, the mean starch content across the varieties/lines significantly decreased by 42% in the controls and 51% in the stressed plants. At the milky stage, the starch content returned to the original level and remained high until maturity. Notably, the starch contents in the stressed flag leaves of KDML105 tended to be higher than the others at all stages. Therefore, the total and non-reducing sugars in flag leaves tended to continuously increase with age in both non-stressed and stressed plants while the mean starch contents were not significantly different among age groups, except for that at the flowering stage, which was significantly lowered.

#### 2.3.5. Sodium (Na^+^) and Potassium (K^+^) Ions

An analysis of the Na^+^ and K^+^ in the roots and leaves of rice plants at harvest revealed a 4- to 5-fold increase in Na^+^ in the roots and between a 14- and 44-fold increase in the leaves of salt-treated plants compared with the controls (Table 4). In response to the salt treatment, K^+^ was slightly reduced in the roots but increased in the leaves. The ratio between Na^+^ and K^+^ (Na^+^/K^+^) increased six- to seven-fold in roots of all three rice varieties/lines. In contrast, the level of increments in Na^+^/K^+^ in the leaf tissues varied from a 10-fold increase in KDML105 to 48-fold in TSKC1-144. The significantly higher Na^+^ content and Na^+^/K^+^ in the leaves of KDML105 compared with those in RD73 and TSKC1-144 indicated the lower capability of Na^+^ exclusion and higher salt susceptibility.

#### 2.3.6. Biomass, Yield, and Yield Components

In this study, NaCl was added to the soils at the early booting stage when the vegetative growth was robust, so the salinity posed no significantly negative effects on the biomass of vegetative organs, including the height, tiller number, root DW, leaf DW, and stem DW (except a significant increase in KDML105), as well as the number and length of the panicles as shown in Table 5. In contrast, salinity caused profoundly damaging effects during flower and seed development. Salt-stressed plants produced a 12–16% reduction in the number of healthy filled seeds panicle^−1^ and a 42–48% increase in the number of poor unfilled seeds panicle^−1^, indicating a hindrance in successful fertilization. Salt stress posed a significant effect on grain development, assimilate translocation, and grain filling, resulting in a significant 5–8% reduction in the 100-seed weight in all three varieties/lines, followed by the significant 19–22% reductions in filled seed weight panicle^−1^. Yields of healthy filled seeds plant^−1^ were reduced by 25–29%, with a significant reduction in RD73. Among the varieties/line, KDML105 suffered the most damage from salt stress, resulting in the lowest number of filled seeds panicle^−1^ (101), the highest and significant increase in unfilled seeds panicle^−1^ (48%), the highest number of unfilled seeds panicle^−1^ (66), the highest and significant increase in unfilled seed weight panicle^−1^ (61%), and, finally, the lowest filled seed weight plant^−1^ (16.91 g). A comparison between RD73 and TSKC1-144 revealed that RD73 had a lower number of healthy filled seeds panicle^−1^ (125 cf. 139), lower filled seed weight panicle^−1^ (2.94 cf. 3.34 g), and lower 100-seed weight (2.36 cf. 2.58 g). However, RD73 produced nine panicles plant^−1^, while TSKC1-144 had only six, finally resulting in RD73 having a higher filled seed weight plant^−1^ (27.57 g) compared with TSKC1-144, which produced 20.93 g filled seed weight plant^−1^.

### 2.4. Correlation Among Physiological Characters of Flag Leaves, Biomass, and Yields

**Figure 7:**
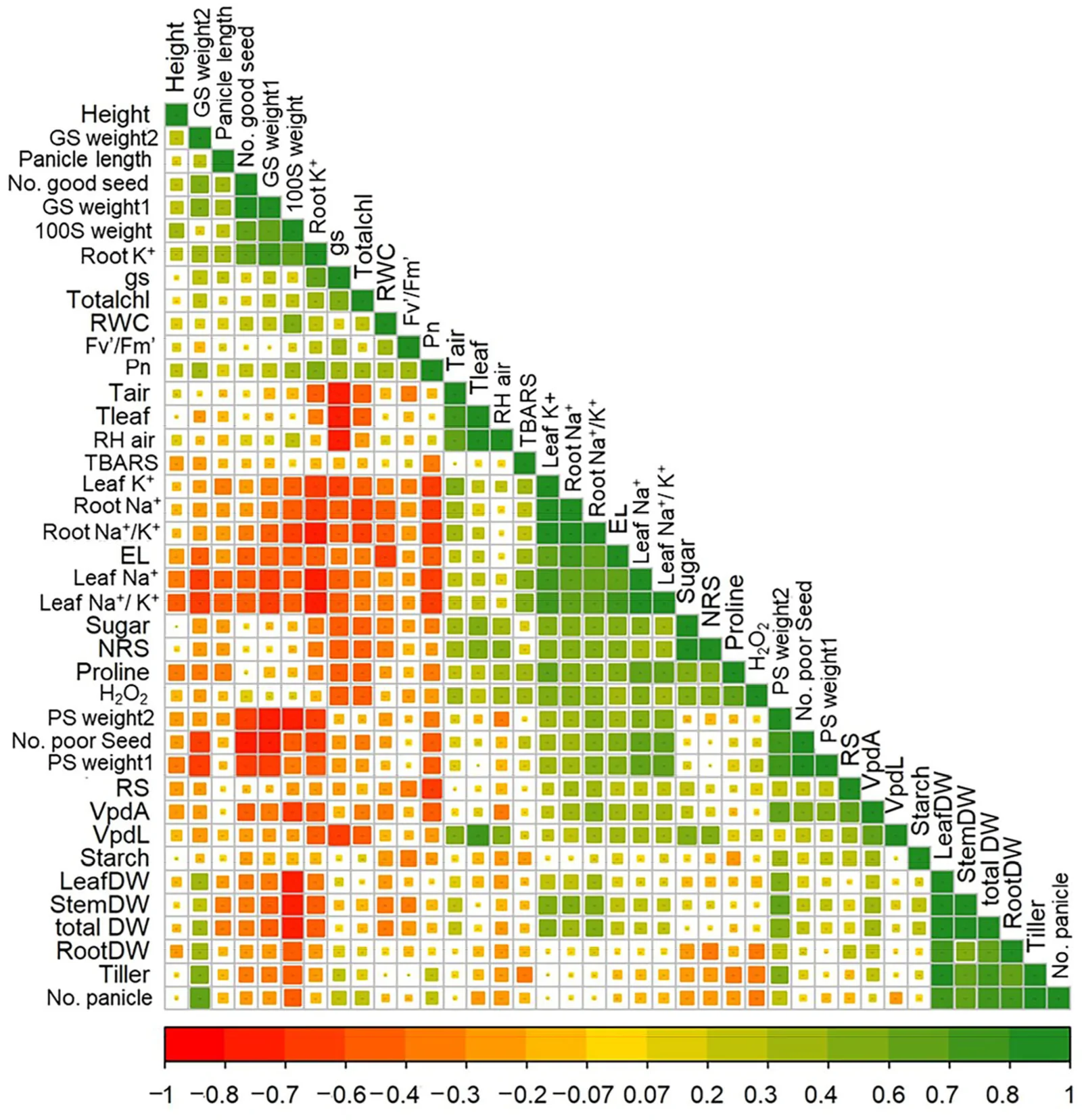
A correlation matrix of physiological/biochemical characters of flag leaves of three rice varieties/line at different stages from booting to maturity, temperature and humidity data during leaf gas exchange measurement, biomass and ion contents at harvest, and yield/yield components.

The correlation matrix depicted in Figure 7 was generated from the physiological parameters of flag leaves during the flowering to mature stages, together with the grain yield data, biomass and ion contents in leaves and roots at harvest, and temperature and humidity data during the time of leaf gas exchange measurements at five growth stages. The net photosynthesis rate (Pn) of flag leaves was significantly correlated (r = 0.43, *p* ≤ 0.05) to stomatal conductance (gs). In turn, gs was negatively correlated to the air temperature (T_air_), leaf temperature (T_leaf_), air relative humidity (RH_air_), and leaf vapor pressure deficit (VPD_L_), indicating that carbon assimilation depends directly on the environmental factors controlling the stomatal aperture. Pn was also positively correlated with parameters indicative of a healthy leaf status i.e., total chlorophyll (Totalchl; r = 0.40, *p* ≤ 0.05), Fv′/Fm′ (r = 0.43, *p* ≤ 0.05), and relative water content (RWC; r = 0.36, non-significant). In contrast, the reduction in Pn was related to an increase in the biochemical parameters related to cellular damage under stress, including electrolyte leakage (EL; r = −0.55, *p* ≤ 0.05), TBARS (r = −0.43, *p* ≤ 0.05), and proline (r = −0.44, *p* ≤ 0.05). Importantly, Pn showed strong negative correlations with leaf Na^+^ and leaf Na^+^/K^+^, both with the significant (*p* ≤ 0.05) correlation coefficients of −0.69. This indicated that photosynthesis performance was retarded by an imbalance of potassium and sodium ions during salt stress. In contrast, Pn was positively correlated with root K^+^ (r = 0.52; *p* ≤ 0.05), indicating the significant roles of root K^+^ in controlling the water absorption and root-to-shoot ion balance.

The healthy filled seed weight plant^−1^ (GS weight 2), filled seed weight panicle^−1^ (GS weight 1), number of filled seeds panicle^−1^ (No. good seed) and 100-seed weight (100S weight) were positively correlated, although not significantly, with Pn. However, the unfilled poor seed weight panicle^−1^ (PS weight 1) and number of unfilled poor seed panicle^−1^ (No. poor seed) were significantly (*p* ≤ 0.05) negatively correlated with Pn, with correlation coefficients of −0.47 and −0.54, respectively. This indicated that impaired photosynthesis during the reproductive phase was associated with a lowered success of fertilization and grain filling. The good filled seeds plant^−1^ was positively (*p* ≤ 0.05) correlated with the tiller number, panicle number, leaf, stem and total dry weight, and negatively with poor seed weight and number. Important physiological parameters which correlated significantly (*p* ≤ 0.05) and negatively with the good filled seeds plant^−1^ included leaf Na^+^ (r = −0.61), leaf Na^+^/K^+^ (r = −0.64), and EL (r = −0.49). The seed weight (100-seed weight), which reflected the efficient grain filling, plumpness, and high starch content, was negatively and significantly (p ≤ 0.05) correlated with the biomass of the leaf (r = −0.77), stem (r = −0.76), root (r = −0.57), and total plant (r = −0.79), indicating that the grain size was partly associated with the remobilization of stored carbon from the leaf and stem. Physiological parameters which are significantly (*p* ≤ 0.05) and positively correlated with 100-seed weight included RWC (r = 0.53) and root K^+^ (r = 0.72), while EL, and leaf and root Na^+^ and Na^+^/K^+^ were negatively correlated.

### 2.5. Principal Component Analysis (PCA) and Hierarchical Clustering Analysis (HCA)

**Figure 8:**
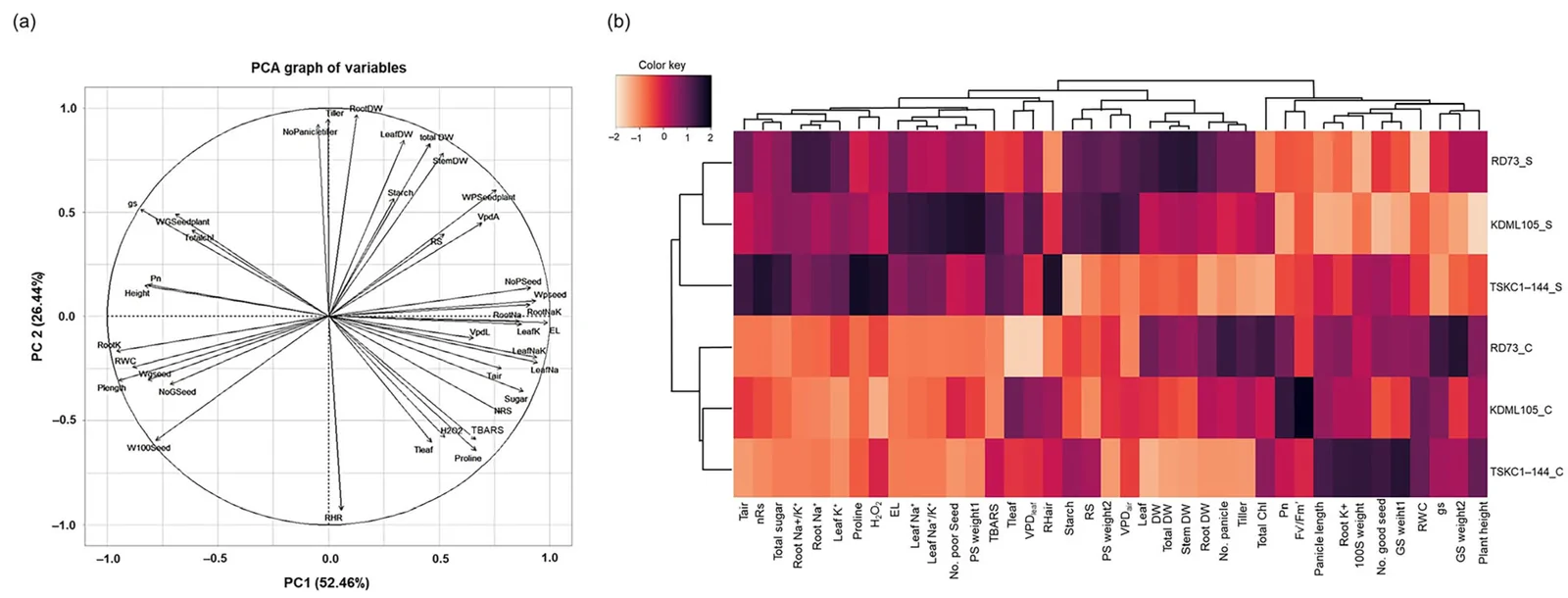
Loading plot of principal component analysis (PCA) of physiological/biochemical parameters of flag leaves at five developmental stages, biomass and ion contents at harvest, and yield components of three rice varieties/line under salt stress (**a**), heatmap depicting the clustering of varieties/lines based on physiological/biochemical parameters of flag leaves at five developmental stages, biomass and ion contents at harvest, and yield components (**b**). For each parameter, the more darkened colors represent the greater, and the lighter the lower values.

To clearly visualize the relationships among physiological variables in response to salt stress and their relation to yield, PCA was performed using physiological parameters of flag leaves at five developmental stages, biomass and ion contents at harvest, and grain yield data, and the loading plot is displayed in Figure 8a. The principal component 1 (PC1) and principal component 2 (PC2) explained 52.46% and 26.44% of the total variation, respectively. On PC1, the physiological parameters that are positively related to number of good filled seeds panicle^−1^ (Nogseed), good seed weight panicle^−1^ (Wgseed), and 100-seed weight (W100seed) included RWC and root K^+^, whereas the net photosynthesis rate (Pn), total chlorophyll (totalchl), and stomatal conductance (gs) were related to the seed weight plant^−1^ (Wgseedplant). Contrastingly, the parameters on PC1 which were positively related to the number of poor unfilled seeds panicle^−1^ (NoPseed) and weight of poor unfilled seeds panicle^−1^ (WPseed) included the root Na^+^, root Na^+^/K^+^, leaf K^+^, leaf Na^+^, leaf Na^+^/K^+^, and EL. Poor seeds also changed in the same direction as H_2_O_2_, TBARS, proline, total sugar, and non-reducing sugar. On PC2, the dry weights of the leaf, stem, root, and total plant, and tiller and panicle number were related and also associated with the number of poor seeds plant^−1^ (WPSeedplant).

The HCA analysis displayed in Figure 8b clearly separated the control and salt-stressed plants into two major groups based on the differences in all physiological and agronomical data. Within the control cluster, most physiological and agronomical characters of TSKC1-144 and KDML105 shared some similarity, and, hence, they are clustered together. The most prominent characters of RD73 that were different from the others included a higher plant biomass, tiller and panicle number, and grain weight plant^−1^. The distinguishing characteristics of TSKC1-144 plants under the non-stressed condition included the long panicle, high good seed weight panicle^−1^, high number of good seeds panicle^−1^, high 100-seed weight, and high root K^+^. Within the salt-stressed group, KDML105 and RD73 shared more similar agronomical and physiological values, and, hence, were grouped together while TSKC1-144 was separated. KDML105 was distinguished by the high number of poor seed panicle^−1^, high poor seed weight panicle^−1^, high poor seed weight plant^−1^, high leaf Na^+^, leaf Na^+^/K^+^, and high EL. RD73 was noted for its high biomass, tiller and panicle number, high good seed weight plant^−1^, and high root Na^+^ and root Na^+^/K^+^. Under salt stress, TSKC1-144 outperformed the others by having a higher number and weight of good seeds panicle^−1^ and higher 100-seed weight, while its distinct flag leaf physiological characters included a high proline, high H_2_O_2_, and high total and non-reducing sugar.

## 3. Discussion

The comparison of physiological responses at different growth stages of the salt-sensitive KDML105 and its genetically improved variety RD73 and line TSKC1-144 revealed different patterns of responses at each growth stage. RD73 is the first genetically bred salt-tolerant rice with a KDML105 genetic background introgressed with *Saltol* QTL, officially released as a new cultivar by the Thailand Rice Department [25]. TSKC1-144 is a backcross pyramided line with a KDML105 genetic background introgressed with a drought-tolerant QTL on chromosome 8 (DT-QTL8) and the *SKC1* salt-tolerant gene on chromosome 1 into KDML105 [27]. At the young seedling stage, KDML105 was highly salt susceptible, showing extremely severe salt injury (mean SES score of 8.55), with near-complete seedling mortality after 15 days of exposure to 150 mM NaCl (Figure 1). The newly improved line TSKC1-144 was highly tolerant at the young seedling stage, able to resist salt stress for 15 days with a slight change in SES scores. It was almost as tolerant as Pokkali and more tolerant than RD73 (Figure 1). An evaluation of the salt tolerance level of hydroponically grown rice at the seedling stage based on the SES scores of the visual salt injury of the seedling shoots [28] has been widely used for decades in numerous studies for the screening and selection of salt-tolerant breeding lines [29] and also for identifying salt-tolerant rice landraces and wild rice [30,31,32]. However, a poor correlation was found between salt tolerance at the seedling stage and that at other stages, especially the reproductive phase [33,34]. As an example, FL478 (a recombinant inbred line derived from Pokkali), which is highly tolerant at the seedling stage, was found to be highly sensitive at the reproductive stage, showing a 96% decrease in the grain number. On the contrary, Sadri, an Iranian variety which is highly sensitive at the seedling stage, became relatively more tolerant than FL478, expressing only a 70% reduction in filled grains [35]. A recent report on QTL mapping using an introgression population derived from a cross between a high-yielding cultivar ‘Cheniere’ and a salt-tolerant donor ‘TCCP’ revealed that salinity tolerance in rice at different growth stages (germination, vegetative, and reproductive) was determined by different mechanisms and different sets of genes/QTLs, and none of the tested introgression lines were highly tolerant at all growth stages [36]. The general cellular mechanisms for salt tolerance included sodium ion exclusion, osmotic tolerance, tissue tolerance, antioxidant defense, and the maintenance of photosynthesis [8,12]. In addition, during the reproductive phase, the ability to protect spikelet sterility and successful fertilization, and maintaining efficient carbohydrate transport from leaves to developing grains are crucial for sustaining grain yields [29,36]. Therefore, the improved breeding lines and potential landraces should be further evaluated for salt tolerance at later growth stages. In this study, it should be noted that the seedling-stage experiment was conducted in a hydroponic culture, whereas the vegetative- and reproductive-stage experiments were conducted in soil. While the NaCl concentration was identical, the actual stress intensity perceived by the roots may differ between the two systems. Therefore, direct quantitative comparisons across stages should be interpreted with caution.

At the vegetative stage, the significant reduction in root length and shoot height of all rice varieties/line subjected to 150 mM NaCl stress for nine days (Table 3) indicated a strong inhibition of cell elongation and expansion as a result of the retardation of root water uptake and loss of cell turgor which is required for cell division and expansion [9,37]. In addition, the high Na^+^ in the apoplast inhibits cell wall loosening by interacting with negatively charged cell wall polymers, hindering the cell’s ability to extend its cell wall, thereby restricting growth [38]. The accumulation of Na^+^ also enhanced leaf senescence, resulting in more yellowing and dead leaves and fewer green photosynthesizing leaves. Biomass reduction in the stressed plants (Table 3) was driven by three interconnected physiological processes; growth restriction, accelerated leaf senescence, and the reduction in CO_2_ assimilation [12]. An exposure to salinity stress for three to nine days caused a significant reduction in the net photosynthesis rates (Pn) of the surviving leaves, particularly KDML105 (Table 1). A previous experiment at the vegetative stage also found more reduction in the Pn of KDML105 than some of the more tolerant varieties [39]. The major inhibitory effects of salt stress on photosynthesis were related to stomatal closure due primarily to osmotic stress, and, in the longer term, ionic stress, which limited CO_2_ access to the chloroplasts [7,33]. Furthermore, rice varieties at the vegetative stage with a better osmotic adjustment ability were found to maintain a higher Pn under salt stress [11]. In addition, ion toxicity (due to toxic Na^+^) and oxidative stress (due to ROS) damaged the chloroplast structural and functional integrity, leading to a reduced PSII photochemical efficiency and the inhibition of Calvin cycle enzymes [14]. The high concentration of Na^+^ in leaf tissues also disrupted the membrane integrity, resulting in increased electrolyte leakage (EL) (Table 1), particularly in KDML105. Membrane damage was done primarily through the displacement of Ca^2+^ by excessive Na^+^ and, secondarily, by the Na^+^-induced ROS accumulation and lipid peroxidation of membrane lipids [40]. The significant accumulation of starch in the leaves of KDML105 and RD73 (Table 1) under salt stress reflected reduced assimilate partitioning due to the restricted growth (Table 3); hence, photosynthetic products were stored as starch [41,42]. Starch, considered a key determinant of plant fitness under abiotic stress, may be remobilized and utilized as an energy source during and after recovery from stress [43].

Considering the differences in the physiological responses among the three varieties/lines, it could be inferred that KDML105 experienced more damages from salinity than RD73 and TSKC1-144 based on the higher percentage reduction in Pn and leaf RWC, the highest leaf EL, the highest leaf Na^+^/K^+^, and, particularly, the higher percentage reduction in the shoot and total dry weight. The lower Na^+^ and Na^+^/K^+^ in leaves of RD73 and TSKC1-144 compared with that in KDML105 (Table 2) reflected the more efficient Na^+^ exclusion from shoots due to the presence of the introgressed *Saltol* QTL and the *SKC1* gene [25,27]. The significantly lower leaf Na^+^ and Na^+^/K^+^ of TSKC1-144 compared with those of RD73 indicated the more efficient ion exclusion. Furthermore, the high concentration of Na^+^ and the high Na^+^/K^+^ in roots of TSKC1-144 indicated that this line was the most efficient in root Na^+^ sequestration into vacuoles. This was consistent with previous reports that salt-tolerant varieties had a greater capability for root Na^+^ sequestration, root K^+^ retention, and root tissue tolerance [13,44].

Salt tolerance at the reproductive stage is crucial for rice growing in the salt-affected soil because it determines the yield and quality of the harvested grains. In this study, NaCl solution was added to the pots at the early booting stage approximately two weeks after the plants have reached maximum vegetative growth; therefore, the biomass of all vegetative parts of the stressed rice was not reduced. Instead, the total plant dry weight of stressed plants was significantly increased mainly as a result of the increased stem dry weight (Table 5). Under salt stress, the stem and leaf sheaths act as a temporary sink that sequesters toxic sodium ions so that less harmful ions reach the actively photosynthesizing leaves. In a previous study, it was reported that the increased biomass of stem and leaf sheath could support higher sodium ion accumulation, and less toxic ions would be transported to the leaves [12].

In a previous study, an analysis of Na^+^ in individual leaves of rice plants subjected to salt stress for 10 days revealed that mature leaves of rice acted as the active sink for Na^+^, with the lowest leaf (the fourth leaf below the flag leaf) accumulating the highest content of Na^+^ (4.0 mmol g^−1^ DW), with the upper leaves containing sequentially less Na^+^ and the flag leaf containing the lowest Na^+^ at 0.025 mmol g^−1^ DW [35]. The effective exclusion of toxic Na^+^ from flag leaves resulted in marginal physiological damages, including a non-significant reduction in chlorophyll contents (Figure 3a), similar to a previous report on five Thai rice varieties [45]. Consequently, the chlorophyll fluorescence parameters were not significantly reduced, indicating the functional sustainability of PSII efficiency and electron transport activity (Figure 3b). Flag leaves were also efficient in maintaining the RWC through the reduction in the transpiration rate due to stomatal closure [46]. As a consequence of the well-maintained water status and low level of oxidative stress (non-significant increase in H_2_O_2_ and lipid peroxidation), the membrane integrity was stabilized until the mature stage when the EL was significantly increased, with KDML105 membrane being the most damaged (Figure 4b).

While Na^+^ sequestration by old leaves protected many physiological traits of flag leaves from being damaged by salt stress [35], the amount of Na^+^ reaching the flag leaves was toxic enough to induce a significant reduction in CO_2_ assimilation, leading to significant reductions in Pn (Figure 3c). This implies that the yield of rice can potentially be enhanced by sustaining the photosynthesis capacity during grain filling stages [47]. The most important factors retarding photosynthesis were the leaf Na^+^ and leaf Na^+^/K^+^, which showed a high negative correlation with Pn (Figure 7). The direct ionic effect on stomatal closure was demonstrated by [48], whereby, under salt stress, the Na^+^ concentration in the apoplast around leaf cells, including guard cells, increased more than 100-fold, thus strongly inducing water loss from guard cells, leading to stomatal closure. However, the observation that the intercellular CO_2_ concentration (Ci) was not significantly reduced upon salt stress suggested that metabolic functions inside the chloroplasts relating to CO_2_ assimilation could also be restricted [49]. The photosynthesis of flag leaves was positively correlated with physiological parameters indicative of healthy leaves, including RWC, chlorophyll content, and Fv′/Fm′, but negatively correlated with cellular damage indicators like EL and TBARS (Figure 7). Among the varieties/lines, the Pn of KDML105 under stress was lower than the others and was significantly reduced at all stages (Figure 3c), indicating that salt stress posed the most adverse effects on the CO_2_ assimilation of KDML105 (Table 4). The higher sensitivity of photosynthesis to the salt stress of KDML105 compared with the improved cultivars was also previously reported [7,38]. The stronger inhibitory effects of salt stress on the photosynthesis of KDML105 was related to its higher Na^+^ and Na^+^/K^+^ in the leaves compared with those in RD73 and TSKC1-144 (Table 4). It should be noted that the Na^+^ exclusion ability of RD73 and TSKC1-144 varied with the developmental stage. At the vegetative stage, the leaf Na^+^ concentration of RD73 and TSKC1-144 were 1.94% and 1.32%, respectively (Table 2), while the values at the reproductive stage were 0.86% for RD73 and 1.34% for TSKC1-144 (Table 4). The variability of the Na^+^ exclusion activity during different growth stages was demonstrated to be associated with the variation in the *OsHKT1;5* (or *SKC1*) mRNA expression in the roots and basal stem of a japonica rice cultivar Nipponbare [50]. Therefore, understanding the genotypic and stage-specific differential Na^+^ exclusion capacity may help farmers make strategic decisions on the selection of varieties to cultivate based on specific field conditions.

While salt stress applied during the reproductive phase did not inhibit growth and biomass of the vegetative parts, it severely affected fertilization, seed setting, photosynthesis, source–sink translocation, grain development, and filling [44,51]. Salt stress caused an impairment in photosynthesis, leading to a significant reduction in the panicle dry weight, resulting from the failure in fertilization and retardation in grain filling [52], as evidenced by the negative correlations between Pn and the number and weight of poor unfilled seeds (Figure 7). The highest reduction in the number of filled seeds panicle^−1^ and the highest increase in the number and weight of unfilled seeds panicle^−1^ occurred in KDML105 (Table 5). The low 100-seed weight of KDML105 also indicated the low efficiency of the grain-filling process mainly related to its lower Pn during the flowering to mature stage. The lower grain yield of KDML105 might also be related to a restriction in assimilate partitioning as shown by the tendency for an increased starch concentration in flag leaves under stress, and the higher starch accumulation from the flowering to mature stage compared with RD73 and TSKC1-144 (Figure 6d; Table S5). Salt stress effects on assimilate partitioning in KDML105 were also evident at the vegetative stage, as shown by its highest percentage increase in leaf starch content (Table 1), consistent with the highest percentage biomass reduction (Table 3). On the other hand, the fertilization and grain-filling process of TSKC1-144 was the least affected, resulting in the highest number and weight of the filled seeds panicle^−1^, the lowest number of the unfilled seeds panicle^−1^ and the highest 100-seed weight (Table 5). However, the final seed yield plant^−1^ of TSKC1-144 was lower than that of RD73 because it produced a much lower number of tillers and panicles per plant, which could be associated with the introgression of DT-QTL8 or with the linkage drag from the *SKC1* gene. Considering its high salt tolerance ability due to an efficient Na^+^ exclusion and the favorable agronomic traits of high seed number panicle^−1^ and high 100-seed weight under both control and salt stressed condition, the final yield of TSKC1-144 could be greatly enhanced by increasing its tillering capacity. The increased tillering of rice can potentially be achieved through molecular-assisted breeding through an introgression of genes encoding positive regulators of axillary bud initiation and outgrowth such as *OsMOC1*, *OsMOC3*, *OsNAL1*, etc. [53,54], or genome editing to suppress the negative regulators of tillering such as *OsTB1*, *OsD53*, *OsFON1*, etc. [55]. Alternatively, the foliar application of humic biostimulants [56] and tiller-specific compound fertilizer containing multiple micro- and trace elements [57] were found to increase tiller formation and fertile panicles.

The improved cultivar RD73 and line TSKC1-144 were more tolerant to salinity than the elite cultivar KDML105 at all growth stages, and, therefore, can be recommended for growing in salt-affected areas where rice may be challenged with salinity at different periods throughout the growing season. Salt tolerance at the seedling stage was of paramount importance, especially for direct-seeded rice cultivation where the seeds germinate directly in the upper, salt-accumulating soil layer [58]. With rain-fed transplanting cultivation where 15-to-30-day-old rice seedlings are transplanted into low salinity rain-flooded soils, rice plants during the vegetative stage may encounter heightened salinity stress when a dry spell occurs after transplantation [6]. Salt tolerance during the reproductive phase is the most critical and directly determines the final yield. In rain-fed low land rice cultivation, if the rainy season becomes shortened in drought years, rice at the panicle initiation, flowering, and grain-filling stage will experience increased soil salinity, which disrupts seed setting and grain filling, leading to crop failure [29,48]. Therefore, RD73 and TSKC1-144, with their superior Na^+^ exclusion mechanism, are suitable for cultivation in semi-dry drought-prone salt-affected ecosystems, where rice cultivation depends solely on rainfalls in northeast Thailand and in similar ecosystems. Specifically, TSKC1-144, with its superior tolerance at the seedling stage, could be prioritized for direct seeding cultivation, while RD73 could be chosen for rain-fed transplanting cultivation. These improved varieties/lines not only produce a higher grain yield under salinity than the elite rice, KDML105, but also exhibit similar agronomic traits and grain-cooking quality [25,27].

## 4. Materials and Methods

### 4.1. Plant Materials and Treatments

Seeds of KDML105 were kindly provided by Khon Kaen Rice Research Center, RD73 by Nakorn Rachasima Rice Research Center, TSKC1-144 by Salt Tolerant Rice Research Group, Department of Biology, Khon Kaen University, IR29 (salt susceptible check), and Pokkali (salt tolerance check) by the Department of Agronomy, Khon Kaen University. Three independent experiments were conducted to evaluate salt tolerance at the seedling, vegetative, and reproductive stages using separate batches of plants. All the experiments were conducted under greenhouse conditions at the Department of Biology, Faculty of Science, Khon Kaen University, Thailand. The seedling stage experiment was performed in a hydroponic system for rapid and uniform salt exposure, whereas the vegetative and reproductive stage experiments were conducted in soil-filled pots to simulate field conditions. For the evaluation of salt tolerance at seedling stage, seeds of KDML105, RD73, TSKC1-144, IR29, and Pokkali were surface-sterilized by soaking in 5% sodium hypochlorite, thoroughly rinsed with distilled water, and germinated on moistened germination paper for 2–3 days. Germinated seedlings (10 seedlings/variety or line/replication) were grown for 3 days on plastic grids floated on distilled water in eight plastic containers, each containing 15 L of water. After 4 days, distilled water was replaced with nutrient solution [59]. When the seedlings were 14 days old, salt stress was introduced by replacing nutrient solution in four containers, with the one containing 150 mM NaCl, while the other four containers were maintained in normal nutrient solution as controls. The experimental containers were arranged in RCBD with four replications. The seedlings were evaluated for salt injury and assigned SES scores from 1 to 9 according to [28] for a total of 15 days. During the experimental period, the average daytime air temperature, relative humidity, and mean daily solar radiation inside the greenhouse were 23.7 ± 1.81 to 37.3 ± 2.92 °C, 42.92 ± 8.52 to 84.04 ± 10.34%, and 448 ± 86 µmol photon m^−2^ s^−1^, respectively.

At the vegetative stage, a pot experiment was set up to evaluate the physiological responses of rice subjected to salt stress. Seeds of KDML105, RD73, and TSKC1-144 were germinated as described above. Four 5-day-old seedlings were transferred to each plastic pot containing 10 kg paddy soil flooded with tap water. Each pot represented one experimental unit containing four plants, and four pots were prepared for each genotype × treatment combination, resulting in four biological replicates. Rice plants were grown under greenhouse conditions at the Department of Biology, Faculty of Science, Khon Kaen University. When the plants were 30 days old, the flooded water was decanted and the soils were left to dry for two days. After that, the salt-stressed pots were filled with 150 mM NaCl, flooded to the level 5 cm above soil surface. The electrical conductivity of the flooded solution was regularly maintained at 12–15 dS m^−1^. The control set of pots continued to be flooded with tap water. The pots were arranged in RCBD with four replications. On day 3, 6, and 9 after salt treatment, leaf gas exchange measurements were conducted on the fully expanded leaves. On day 9, physiological traits including the relative water content (RWC) and electrolyte leakage (EL) were investigated on the freshly harvested fully expanded leaves, while biochemical analyses were measured in leaves frozen at −20 °C. During the vegetative stage experimental period, the average daytime air temperature, relative humidity, and mean daily solar radiation inside the greenhouse were 23.7 ± 3.14 to 34.1 ± 1.01 °C, 59.36 ± 14.95 to 97.18 ± 4.48%, and 364 ± 85 µmol photon m^−2^ s^−1^, respectively.

For the reproductive stage experiment, seeds of KDML105, RD73, and TSKC1-144 were surface-sterilized and germinated as described above. Germinated seedlings were grown in plastic pots as described above using two plants per pot. Each pot represented one experimental unit containing two plants, and four pots were prepared for each genotype × treatment combination, resulting in four biological replicates. Rice plants were maintained under the same greenhouse conditions as described for the vegetative-stage experiment. When the plants reached the early booting stage, i.e., the developing panicles in the main culms were approximately 1–2 cm long, the flooded water was removed and the soils were left to dry partially for two days. After that the pots for salt stress treatments were filled with 150 mM NaCl solution to the level 5 cm above soil surface (day 0), while the control pots were refilled with tap water. The electrical conductivity of the flooded solution was regularly maintained at 12–15 dS m^−1^. The pots were arranged in RCBD with four replications. Physiological responses of the flag leaf were then investigated at the following five developmental stages: (1) late booting stage (day 8–10), (2) flowering stage (day 16–18), (3) milky stage (day 23–24), (4) dough stage (day 30–31), and (5) mature stage (day 34–35). Net photosynthetic rate was measured between 09:00 and 12:00 AM. Fresh leaf samples were collected to determine relative water content (RWC), chlorophyll content, and electrolyte leakage (EL). Additional flag leaf samples were immediately frozen in liquid nitrogen and stored at −20 °C for subsequent physiological and biochemical analyses, including the following: (1) thiobarbituric acid reactive substances (TBARSs), (2) hydrogen peroxide (H_2_O_2_) content, (3) proline content, (4) soluble sugar contents, and (5) starch content. At harvest (5–7 days after mature stage), fresh and dry weights of roots, stems, and leaves were recorded. Oven-dried root and leaf samples were used to determine sodium (Na^+^) and potassium (K^+^) ion contents. Finally, grains were harvested to evaluate yield-related traits. During the reproductive stage experimental period, the average daytime air temperature, relative humidity, and mean daily solar radiation inside the greenhouse were 26.05 ± 1.61 to 33.67 ± 1.72 °C, 59.91 ± 8.91 to 98.24 ± 4.06%, and 402 ± 88 µmol photon m^−2^ s^−1^, respectively.

### 4.2. Chlorophyll Contents and Photosynthesis Performance

Chlorophyll content was determined by extracting pigments from 0.1 g of fresh leaf tissue in 80% acetone. The samples were placed in screw–cap test tubes, tightly sealed, and incubated at room temperature for 48 h or until complete pigment extraction was achieved. The absorbance of the chlorophyll extract was then measured at wavelengths of 645 and 663 nm using a spectrophotometer (model i3, Hanon Instruments Co., Jinan city, Shandong, China), with 80% acetone used as the blank. Chlorophyll a, chlorophyll b, and total chlorophyll contents were calculated according to the equations described by [60].

Net photosynthesis rate (Pn), stomatal conductance (gs), and transpiration rate (Tr) were measured using an LI–6400XT Portable Photosynthesis System (LI–COR Inc., Lincoln, NE, USA). Measurements were conducted under a photosynthetic photon flux density of 1200 µmol m^−2^ s^−1^, CO_2_ concentration maintained at 400 ppm, relative humidity between 30 and 70%, and leaf chamber temperature maintained between 30 ± 2 °C. Data were recorded when the percentage of total coefficient of variation % total CV) was ≤0.2. Measurements were taken at the midpoint of the leaf blade and performed between 09:00 and 12:00 AM.

### 4.3. Relative Water Content (RWC)

The RWC was determined from the middle part of the leaf blade by cutting a 2 cm segment, immediately placed in a pre-weighed micro-tube, sealed, and placed in a box. The tube was weighed to obtain the fresh weight (FW). The leaf segment was then floated on deionized water in a Petri dish placed under fluorescent light for 4 h at room temperature. Then, the leaf sample was re-weighed to obtain the turgid weight (TW), and then dried in an oven at 80 °C for 48 h for determination of dry weight (DW). The relative water content (RWC) was calculated following [61] using the following equation:

RWC (%) = [(FW − DW)/(TW − DW)] × 100

### 4.4. Electrolyte Leakage

Electrolyte leakage (EL), an indicator of membrane integrity, was determined according to the method of [62]. Fresh leaf samples (0.1 g) were placed in 15 mL tubes containing 10 mL of deionized distilled water and incubated at room temperature for 4 h. After incubation, the electrical conductivity (EC) of the solution was measured using a conductivity meter (PL–700PCS GOnDO, Taipei City, Taiwan), and the value was recorded as EC1. The samples were then boiled at 100 °C for 30 min to release all electrolytes. After cooling to room temperature, the electrical conductivity was measured again and recorded as EC2. The percentage of electrolyte leakage (EL) was calculated using the following equation:

EL (%) = (EC1/EC2) × 100

### 4.5. Sugar, Starch, and Proline

#### 4.5.1. Extraction of Soluble Sugars and Starch

Extraction of soluble sugars and starch was performed according to the method described by [63]. Fresh leaf tissue (0.1 g) was ground to a fine powder in liquid nitrogen and homogenized with 1 mL of 80% ethanol. The homogenate was centrifuged at 12,000 rpm for 10 min. After centrifugation, the supernatant was carefully collected, and its volume was measured and recorded. The supernatant was stored at −20 °C for subsequent determination of soluble sugar contents. The remaining pellet, along with the recorded volume of the supernatant, was also stored at −20 °C for further analysis of total sugar and starch contents.

#### 4.5.2. Total Sugar

Total soluble sugar content was determined using the anthrone method [63]. An aliquot of 10 µL of the extracted sugar solution was mixed with 90 µL of distilled water and 600 µL of anthrone reagent containing 0.5 mM anthrone dissolved in 70% sulfuric acid. The reaction mixture was incubated in a water bath at 95 °C for 12 min. After incubation, the reaction was rapidly cooled for 5 min to terminate the reaction. The absorbance was measured at 620 nm using a spectrophotometer and compared with a standard curve prepared using glucose. Total soluble sugar content was calculated and expressed as mg g^−1^ fresh weight (FW).

#### 4.5.3. Reducing Sugar

Reducing sugar content was determined according to the method of [64]. The dinitro salicylic acid (DNS) reagent was prepared by dissolving 0.1 mM DNS and 2.5 mM NaOH, followed by mixing in a water bath at 45 °C. Subsequently, 30% potassium sodium tartrate (KNaC_4_H_4_O_6_·4H_2_O) was added, and all components were thoroughly mixed. An aliquot of 30 µL of the sugar extract was allowed to evaporate to dryness, after which 600 µL of distilled water and 300 µL of DNS reagent were added. The reaction mixture was heated in a boiling water bath at 95 °C for 8 min, and then cooled to terminate the reaction. Absorbance was measured at 540 nm using a spectrophotometer and compared with a standard curve prepared using glucose. Non-reducing sugar content was calculated by subtracting the reducing sugar content from the total sugar content, as described by [65].

#### 4.5.4. Starch

Starch content was determined according to the method described by [66]. The pellet obtained from the extraction procedure described in Section 4.5.1 was resuspended in 500 µL of distilled water, followed by the addition of 650 µL of 52% perchloric acid. The mixture was thoroughly homogenized and incubated at room temperature for 30 min. After incubation, the samples were centrifuged at 12,000 rpm for 10 min. An aliquot of 100 µL of the supernatant was diluted with 900 µL of distilled water and mixed thoroughly. Subsequently, 100 µL of the diluted solution was transferred to a new tube and mixed with 500 µL of anthrone reagent containing 0.5 mM anthrone dissolved in 70% sulfuric acid. The reaction mixture was heated at 95 °C for 12 min and then immediately cooled to terminate the reaction. Absorbance was measured at 620 nm using a spectrophotometer. The starch content was calculated by comparison with a standard curve prepared using glucose standards.

#### 4.5.5. Proline

Proline content was analyzed by the modified ninhydrin method following [67] using 0.1 g of leaf tissues. The contents of proline were evaluated based on a standard curve and expressed as μg g^−1^ FW.

### 4.6. Hydrogen Peroxide (H_2_O_2_) and Thiobarbituric Acid Reactive Substances (TBARSs)

Hydrogen peroxide (H_2_O_2_) content was determined according to the method of [68]. Fresh leaf tissue (0.1 g) was ground in liquid nitrogen and homogenized with 1 mL of 0.1% trichloroacetic acid (TCA). The homogenate was centrifuged at 12,000 rpm for 15 min at 4 °C. After centrifugation, 0.5 mL of the supernatant was mixed with 0.5 mL of 10 mM potassium phosphate buffer (K_2_HPO_4_, pH 7.0) and 1 mL of 1 M potassium iodide (KI). The absorbance of the reaction mixture was measured at 390 nm using a spectrophotometer. Hydrogen peroxide content was calculated by comparison with a standard curve generated using known concentrations of H_2_O_2_.

Thiobarbituric acid reactive substances (TBARSs) were determined using the thiobarbituric acid assay described by [68]. Fresh leaf tissue (0.1 g) was homogenized with 1.5 mL of distilled water. Subsequently, 1.5 mL of 0.5% (*w*/*v*) 2-thiobarbituric acid (TBA) prepared in 20% trichloroacetic acid (TCA) was added to the homogenate. The mixture was heated in a boiling water bath for 25 min, and then immediately cooled in an ice bath for 5 min to terminate the reaction. The absorbance of the supernatant was measured at 532 and 600 nm using a spectrophotometer. A blank solution containing 1.0 mL of TBA reagent mixed with 0.5 mL of 0.1% TCA was used for calibration. The TBARS concentration was calculated using an extinction coefficient of 155 mM^−1^ cm^−1^ according to the following equation:

C = A/(E × L),

where C represents the TBARS concentration, A is absorbance at 532 nm—absorbance at 600 nm, E is the extinction coefficient = 155 mM^−1^ cm^−1^, and L is the light path = 1 cm. The results were expressed based on the fresh weight of the samples.

### 4.7. Sodium and Potassium Ions

To determine the concentration of Na and K ions, dried leaves and root samples, approximately 0.1 g each, were ground to fine powder and digested with a solution consisting of 10 mL of nitric acid (300 °C), 5 mL of perchloric acid (200 °C), and 20 mL of 6M hydrochloric acid. The concentrations of Na and K ions were analyzed using an atomic absorption spectrophotometer (932AAA, GBC Scientific, Braeside, VIC, Australia).

### 4.8. Biomass and Yield

Rice cultivation was conducted using the same procedure described in Section 4.1 under greenhouse conditions until the plants reached the harvest stage. Yield performance and yield components were then evaluated, including the number of tillers plant^−1^, plant height, panicle length, number of panicles plant^−1^, number of filled grains panicle^−1^, number of unfilled grains panicle^−1^, weight of filled grains panicle^−1^, weight of unfilled grains panicle^−1^, 100-grain weight (filled grains), total weight of filled grains plant^−1^, total weight of unfilled grains plant^−1^, and dry weights of stems, leaves, and roots.

### 4.9. Statistical Analysis

Data were analyzed by analysis of variance (ANOVA) based on a randomized complete block design (RCBD) with four replications. When significant differences were detected (*p* ≤ 0.05), mean comparisons were performed using the least significant difference (LSD) test to evaluate the effects of salt treatments, rice varieties, and their interactions on physiological biochemical traits, biomass, and yield. Data are presented as the mean ± standard error (SE). Statistical analyses were performed using Statistix version 10 (Analytical Software, Tallahassee, FL, USA). For the reproductive stage experiment, principal component analysis (PCA) and hierarchical cluster analysis (HCA), combined with heatmap visualization, were performed to classify rice varieties grown under different salinity treatments based on plant growth traits, yield components, and physiological and biochemical traits. PCA and HCA were conducted using R version 4.5.2 [69] and RStudio version 2023.12.1 Build 402 [70].

## 5. Conclusions

Sodium chloride stress at 150 mM for 15 days at the young seedling stage resulted in almost all seedling mortality in the salt-sensitive KDML105 rice while the improved variety, RD73, and line TSKC1-144, still survived with some green surviving leaves. Salt stress during the vegetative stage created the most adverse effects on KDML105 based on the highest reduction in biomass, Pn, leaf RWC, and membrane stability. When salt stress was introduced at the early booting stage after the maximum vegetative growth, biomass at harvest of the vegetative parts of all three rice varieties/lines were not affected. Moreover, the physiology of flag leaves, including the chlorophyll content, PSII efficiency, RWC, EL, proline, H_2_O_2_, and TBARS, was hardly affected by salinity except at the mature stage. The most prominent adverse effects of salt stress were evident in the accumulation of leaf Na^+^, the increased leaf Na^+^/K^+^, and the reduction in the Pn of flag leaves with the most inhibitory effects on KDML105. Salt-induced effects on fertilization and the grain-filling process resulted in a significant reduction in the filled grain number and weight per panicle, and 100-grain weight leading to a reduced grain weight per plant with the greatest reduction in KDML105. The improved line TSKC1-144 was more tolerant than the improved variety RD73 at the young seedling stage. Their level of tolerance was similar at the vegetative stage, but RD73 was more tolerant at the reproductive stage based on the higher grain weight per plant. The salt tolerance ability of the improved variety/line should be further confirmed in saline fields in several locations with varying degrees of salinity levels throughout rice-growing seasons. The pyramided line TSKC1-144 may be genetically improved for a higher tiller number trait to increase its yield potential. The information from this study could also benefit the planning for soil, water, and cultural management to optimize the growth of specific varieties in saline fields, as salt tolerance fluctuates across the seedling, vegetative, and reproductive stages.

## Figures and Tables

**Table 1:** Chlorophyll content, starch and sugar content, relative water content (RWC), and electrolyte leakage (EL) of rice leaves at the vegetative stage, after being treated with 150 mM NaCl for 9 days from 30 to 39 days after germination, and the net photosynthesis rate (Pn) measured after 3, 6, and 9 days after NaCl exposure.

Variety/Line	Control	Salt	% Change	Control	Salt	% Change
	Total chlorophyll (mg g^−1^ FW)			Pn—day 3 (µmol CO_2_ m^−2^ s^−1^)		
KDML105	2.65 ^a^	2.86 ^a^	8	21.53 ^a^	8.13 ^b^	−62 *
RD73	3.14 ^a^	3.25 ^a^	3	19.03 ^a^	7.25 ^b^	−62 *
TSKC1-144	3.21 ^a^	3.37 ^a^	5	19.60 ^a^	7.31 ^b^	−63 *
mean	3.00	3.16	5.33	20.05	7.56	−62.33
	Pn–day 6 (µmol CO_2_ m^−2^ s^−1^)			Pn—day 9 (µmol CO_2_ m^−2^ s^−1^)		
KDML105	18.63 ^a^	6.54 ^b^	−65 *	17.38 ^a^	8.85 ^b^	−49 *
RD73	18.80 ^a^	8.76 ^b^	−53 *	18.33 ^a^	12.15 ^ab^	−34
TSKC1-144	18.60 ^a^	8.98 ^b^	−52 *	18.68 ^a^	10.45 ^b^	−44 *
mean	18.68	8.09	−56.67	18.13	10.48	−42.33
	Starch (mg g^−1^ DW)			Total sugars (mg g^−1^ DW)		
KDML105	55.94 ^c^	69.00 ^ab^	23 *	66.92 ^a^	81.28 ^a^	21
RD73	63.64 ^bc^	75.69 ^a^	19 *	78.09 ^a^	66.98 ^a^	−14
TSKC1-144	74.55 ^ab^	77.19 ^a^	4	80.08 ^a^	68.62 ^a^	−14
mean	64.71	73.96	14	75.03	72.29	−4
	Non-reducing sugar (mg g^−1^ DW)			Reducing sugar (mg g^−1^ DW)		
KDML105	57.73 ^a^	69.32 ^a^	20	9.16 ^a^	11.96 ^a^	30
RD73	58.94 ^a^	61.44 ^a^	4	10.09 ^a^	7.63 ^a^	−24
TSKC1-144	63.04 ^a^	61.35 ^a^	−3	12.01 ^a^	7.61 ^a^	−37
mean	59.90	64.04	7	10.43	9.07	−13
	Relative water content (%)			Electrolyte leakage (%)		
KDML105	96.3 ^a^	88.0 ^cd^	−9 *	3.754 ^b^	6.252 ^a^	67 *
RD73	93.5 ^ab^	86.8 ^d^	−7 *	3.569 ^b^	4.767 ^b^	34
TSKC1-144	93.0 ^ab^	90.3 ^bc^	−3	3.170 ^b^	3.721 ^b^	17
mean	94.27	88.37	−6.33	3.50	4.91	39.33

Note: Significant differences (*p* ≤ 0.05) among varieties/line and growth conditions are indicated with different lowercase letters; * indicated a significant difference (*p* ≤ 0.05) between the control and the salt stress condition of each variety/line.

**Table 2:** Sodium ions (Na^+^), potassium ions (K^+^), and ratio between sodium and potassium ions (Na^+^/K^+^) in leaf and root tissues of three rice varieties/lines exposed to salinity stress (150 mM NaCl) for 9 days at the vegetative stage, from 30 to 39 days after germination.

Parameter	Variety/Line	Control	Salt	Fold Change
Leaf Na^+^	KDML105	0.07 ^c^	1.81 ^a^	25.86 *
	RD73	0.08 ^c^	1.94 ^a^	24.25 *
	TSKC1-144	0.03 ^c^	1.32 ^b^	40.62 *
	mean	0.06	1.69	30.24
Leaf K^+^	KDML105	2.95 ^ab^	2.33 ^b^	0.79
	RD73	3.39 ^a^	2.75 ^ab^	0.81
	TSKC1-144	2.86 ^ab^	3.38 ^a^	1.18
	mean	3.07	2.82	0.93
Leaf Na^+^/K^+^	KDML105	0.02 ^c^	0.94 ^a^	38.16 *
	RD73	0.02 ^c^	0.71 ^a^	35.38 *
	TSKC1-144	0.01 ^c^	0.39 ^b^	31.20 *
	mean	0.017	0.68	34.91
Root Na^+^	KDML105	0.24 ^c^	1.61 ^ab^	6.61 *
	RD73	0.30 ^c^	1.45 ^b^	4.87 *
	TSKC1-144	0.30 ^c^	1.74 ^a^	5.85 *
	mean	0.28	1.60	5.78
Root K^+^	KDML105	1.04 ^ab^	0.72 ^bc^	0.69
	RD73	1.20 ^a^	0.78 ^bc^	0.65 *
	TSKC1-144	0.86 ^bc^	0.64 ^c^	0.74
	mean	1.03	0.71	0.69
Root Na^+^/K^+^	KDML105	0.26 ^d^	2.23 ^b^	8.72 *
	RD73	0.25 ^d^	1.90 ^c^	7.68 *
	TSKC1-144	0.36 ^d^	2.76 ^a^	7.71 *
	mean	0.29	2.30	8.04

Note: Significant differences (*p* ≤ 0.05) among varieties/lines and growth conditions are indicated with different lowercase letters; * indicated a significant difference (*p* ≤ 0.05) between the control and the salt stress condition of each variety/line.

**Table 3:** Growth parameters of three rice varieties/lines at the vegetative stage exposed to 150 mM NaCl for 9 days from 30 to 39 days after germination.

Parameter	Variety/Line	Control	Salt	% Change
Root length (cm)	KDML105	26.53 ^a^	16.90 ^b^	−36 *
	RD73	26.05 ^a^	16.63 ^b^	−36 *
	TSKC1-144	28.65 ^a^	14.75 ^b^	−49 *
	mean	27.08	16.09	−40
Plant height (cm)	KDML105	102.98 ^a^	82.82 ^c^	−20 *
	RD73	99.73 ^a^	75.68 ^d^	−24 *
	TSKC1-144	103.30 ^a^	88.13 ^b^	−15 *
	mean	102.00	82.21	−19
Shoot FW (g)	KDML105	13.23 ^ab^	8.15 ^c^	−38 *
	RD73	11.68 ^b^	5.40 ^d^	−54 *
	TSKC1-144	14.98 ^a^	9.55 ^c^	−36 *
	mean	13.30	7.70	−48
Root FW (g)	KDML105	5.23 ^b^	2.70 ^c^	−48 *
	RD73	6.60 ^a^	1.65 ^d^	−75 *
	TSKC1-144	7.48 ^a^	2.40 ^cd^	−68 *
	mean	6.44	2.25	−63
Total FW (g)	KDML105	18.45 ^b^	10.80 ^c^	−41 *
	RD73	18.28 ^b^	7.05 ^d^	−61 *
	TSKC1-144	22.50 ^a^	11.90 ^c^	−47 *
	mean	19.74	9.92	−49
Shoot DW (g)	KDML105	2.83 ^a^	1.45 ^c^	−49 *
	RD73	2.30 ^ab^	1.85 ^bc^	−20
	TSKC1-144	2.83 ^a^	2.15 ^abc^	−24
	mean	2.65	1.82	−31
Root DW (g)	KDML105	0.93 ^a^	0.33 ^b^	−65 *
	RD73	0.93 ^a^	0.23 ^b^	−76 *
	TSKC1-144	0.85 ^a^	0.25 ^b^	−71 *
	mean	0.90	0.27	−70
Total DW (g)	KDML105	3.73 ^a^	1.80 ^c^	−52 *
	RD73	3.23 ^ab^	2.13 ^c^	−34 *
	TSKC1-144	3.70 ^a^	2.40^bc^	−35 *
	mean	3.55	2.11	−40

Note: Significant differences (*p* ≤ 0.05) among varieties/line and growth conditions are indicated with different lowercase letters; * indicated a significant difference (*p* ≤ 0.05) between the control and the salt stress condition of each variety/line.

**Table 4:** Sodium ions (Na^+^), potassium ions (K^+^), and ratio between sodium and potassium ions (Na^+^/K^+^) in root and leaf tissues at harvest of three rice varieties/lines grown under non-saline and saline conditions.

Treatment	Variety/Line	Roots			Leaves		
		Na^+^ (%)	K^+^ (%)	Na^+^/K^+^	Na^+^ (%)	K^+^ (%)	Na^+^/K^+^
Control	KDML105	0.13 ^c^	0.25 ^bc^	0.51 ^c^	0.11 ^d^	1.60 ^c^	0.07 ^d^
	RD73	0.23 ^c^	0.27 ^ab^	0.85 ^c^	0.04 ^d^	2.01 ^b^	0.02 ^d^
	TSKC1-144	0.17 ^c^	0.31 ^a^	0.56 ^c^	0.03 ^d^	1.67 ^c^	0.01 ^d^
	mean	0.18	0.28	0.64	0.06	1.76	0.03
Salt	KDML105	0.67 ^b^	0.17 ^e^	3.88 ^b^	1.77 ^a^	2.51 ^a^	0.71 ^a^
	RD73	1.07 ^a^	0.19 ^de^	5.47 ^a^	0.86 ^c^	2.75 ^a^	0.31 ^c^
	TSKC1-144	0.90 ^a^	0.22 ^cd^	4.01 ^b^	1.34 ^b^	2.76 ^a^	0.48 ^b^
	mean	0.88	0.19	4.45	1.32	2.67	0.50

Note: Significant differences (*p* ≤ 0.05) among varieties/lines and growth conditions are indicated by different lowercase letters.

**Table 5:** Biomass at harvest and yield parameters of three rice varieties/lines grown under non-saline (control) and saline (150 mM NaCl) conditions.

Variety/Line	Control	Stress	% Change	Control	Stress	% Change
	Plant height (cm)			Panicle length (cm)		
KDML105	196 ^a^	188 ^a^	−4	28.73 ^a^	27.79 ^a^	−3
RD73	197 ^a^	196 ^a^	−1	28.74 ^a^	27.98 ^a^	−3
TSKC1-144	198 ^a^	193 ^a^	−3	29.10 ^a^	28.45 ^a^	−2
mean	197	192	−3	28.86	28.07	−3
	Tiller number			Number of filled seeds panicle^−1^		
KDML105	9 ^a^	9 ^a^	−6	120 ^cd^	101 ^d^	−16
RD73	11 ^a^	10 ^a^	−7	144 ^ab^	125 ^bc^	−13
TSKC1-144	6 ^b^	6 ^b^	0	159 ^a^	139 ^bc^	−12 *
mean	9	8	−4	141	122	−14
	Leaf DW plant^−1^ (g)			Number of unfilled seeds panicle^−1^		
KDML105	28.03 ^c^	30.22 ^bc^	8	44.50 ^bc^	66.00 ^a^	48 *
RD73	35.15 ^ab^	37.79 ^a^	8	35.50 ^bc^	51.50 ^ab^	45
TSKC1-144	20.56 ^d^	25.47 ^cd^	24	32.50 ^c^	46.25 ^bc^	42
mean	27.91	31.16	13	38	55	45
	Stem DW plant^−1^ (g)			Filled seed weight panicle^−1^ (g)		
KDML105	75 ^c^	106 ^b^	41 *	3.08 ^c^	2.46 ^d^	−20 *
RD73	111 ^b^	149 ^a^	34	3.64 ^b^	2.94 ^cd^	−19 *
TSKC1-144	54 ^c^	74 ^c^	37	4.29 ^a^	3.34 ^bc^	−22*
mean	80.17	109.84	37	3.67	2.92	−20
	Root DW plant^−1^ (g)			Unfilled seed weight panicle^−1^ (g)		
KDML105	16.95 ^ab^	16.55 ^ab^	−2	0.20 ^b^	0.32 ^a^	61 *
RD73	20.26 ^a^	21.48 ^a^	6	0.17 ^b^	0.24 ^ab^	41
TSKC1-144	10.10 ^b^	9.33 ^b^	−8	0.16 ^b^	0.23 ^ab^	42
mean	15.77	15.79	−1	0.18	0.27	48
	Panicle DW plant^−1^ (g)			100-seed weight (g)		
KDML105	29.66 ^b^	26.00 ^b^	−12	2.57 ^b^	2.43 ^c^	−5 *
RD73	42.87 ^a^	33.62 ^b^	−21 *	2.56 ^b^	2.36 ^d^	−8 *
TSKC1-144	29.66 ^b^	25.12 ^b^	−15	2.72 ^a^	2.58 ^b^	−5 *
mean	34.06	28.25	−17	2.62	2.46	−6
	Total DW plant^−1^ (g)			Filled seed weight plant^−1^		
KDML105	144.10 ^c^	175.74 ^b^	21 *	23.69 bc	16.91 ^c^	−29
RD73	185.22 ^b^	216.65 ^a^	16 *	37.05 a	27.57 ^b^	−26 *
TSKC1-144	120.99 ^c^	127.74 ^c^	5	28.05 ab	20.93 ^bc^	−25
mean	150.1	173.37	−15	29.60	21.81	−27
	Number of panicles plant^−1^			Unfilled seed weight plant^−1^		
KDML105	8 ^bc^	8 ^b–d^	0	1.39 ^b–d^	2.60 ^a^	87 *
RD73	10 ^a^	9 ^ab^	−10	1.45 ^bc^	2.28 ^ab^	57
TSKC1-144	6 ^d^	6 ^cd^	0	0.74 ^d^	1.06 ^cd^	44
mean	8	8	−3	1.19	1.99	63

Note: Significant differences (*p* ≤ 0.05) among varieties/lines and growth conditions are indicated by different lowercase letters; * indicated a significant difference (*p* ≤ 0.05) between the control and the salt stress condition of each variety/line.

## Data Availability

Data can be provided by the authors upon request.

## Supplementary Materials

The following supporting information can be downloaded at https://www.mdpi.com/article/10.3390/plants15172635/s1, Table S1: Total chlorophyll, maximum quantum yield of PS II in the light (Fv′/Fm′), net photosynthesis rate (Pn), stomatal conductance (gs), and intercellular CO_2_ concentration (Ci) of flag leaves of pot-grown rice plants under non-saline (Control) and salt stress (150 mM NaCl) conditions during the reproductive growth stages.; Table S2: Relative water content (RWC) and electrolyte leakage (EL) of flag leaves of pot-grown rice plants under non-saline (Control) and salt stress (150 mM NaCl) conditions during the reproductive growth stages.; Table S3: Hydrogen peroxide (H_2_O_2_), Thiobarbituric acid reactive substances (TBARS), and Proline in flag leaves of pot-grown rice plants under non-saline (Control) and salt stress (150 mM NaCl) conditions during the reproductive growth stages; Table S4: Total sugar, non-reducing sugar (nRS), reducing sugar (RS), and starch content in flag leaves of pot-grown rice plants under non-saline (Control) and salt stress (150 mM NaCl) conditions during the reproductive growth stages; Table S5: Split-plot ANOVA showing levels of significant differences for growth and physiological parameters of rice at the vegetative stage under non-saline (Control) and salt stress (150 mM NaCl); Table S6: Split-plot ANOVA showing levels of significant differences for physiology of flag leaves at different stages, biomass, yield, and yield components of rice at the reproductive stage under non-saline (Control) and salt stress (150 mM NaCl).

## Abbreviations

The following abbreviations are used in this manuscript:

DW	Dry weight
EL	Electrolyte leakage
F_v_′/F_m_′	Maximum quantum yield of PSII in the light
FW	Fresh weight
gs	Stomatal conductance
MABC	Marker-assisted backcross breeding
MDA	malondialdehyde
NRS	Non-reducing sugar
Pn	Net photosynthesis rate
QTL	Quantitative trait locus
ROS	Reactive oxygen species
RS	Reducing sugar
RWC	Relative water content
SES	Standard evaluation system for salt tolerance
TBARSs	Thiobarbituric acid reactive substances

## References

[1] Food and Agricultural Organization of the United Nations (2021). Global Map of Salt-Affected Soils. https://openknowledge.fao.org/items/be52a217-7c1c-41c9-8e7c-c21751f92874.

[2] Li H., Wang S. (2025). Growing risk of soil salinization linked to soil droughts in a changing climate. Geophys. Res. Lett..

[3] Hassani A., Azapagic A., Shokri N. (2020). Predicting long-term dynamics of soil salinity and sodicity on a global scale. Proc. Natl. Acad. Sci. USA.

[4] Arunin S., Pongwichian P. (2015). Salt-affected soils and management in Thailand. Bull. Soc. Sea Water Sci. Jpn..

[5] Arunrat N., Kongsurakan P., Sereenonchai S., Hatano R. (2020). Soil organic carbon in sandy paddy fields of northeast Thailand: A review. Agronomy.

[6] Yang Y., Ye R., Srisutham M., Nontasri T., Sritumboon S., Maki M., Yoshida K., Oki K., Homm K. (2022). Rice production in farmer fields in soil salinity classified areas in Khon Kaen, Northeast Thailand. Sustainability.

[7] Santanoo S., Lontom W., Dongsansuk A., Vongcharoen K., Theerakulpisut P. (2023). Photosynthesis performance at different growth stages, growth, and yield of rice in saline fields. Plants.

[8] Munns R., Tester M. (2008). Mechanisms of salinity tolerance. Annu. Rev. Plant Biol..

[9] Lu Y., Fricke W. (2023). Salt stress-regulation of root water uptake in a whole-plant and diurnal context. Int. J. Mol. Sci..

[10] Singh P., Choudhary K.K., Chaudhary N., Gupta S., Sahu M., Tejaswini B., Sarkar S. (2022). Salt stress resilience in plants mediated through osmolyte accumulation and its crosstalk mechanism with phytohormones. Front. Plant Sci..

[11] Nounjan N., Chansongkrow P., Charoensawan V., Siangliw J.L., Toojinda T., Chadchawan S., Theerakulpisut P. (2018). High performance of photosynthesis and osmotic adjustment are associated with salt tolerance ability in rice carrying drought tolerance QTL: Physiological and co-expression network analysis. Front. Plant Sci..

[12] Sackey O.K., Feng N., Mohammed Y.Z., Dzou C.F., Zheng D., Zhao L., Shen X. (2025). A comprehensive review on rice responses and tolerance to salt stress. Front. Plant Sci..

[13] Liu J., Shabala S., Shabala L., Zhou M., Meinke H., Venkataraman G., Chen Z., Zeng F., Zhao Q. (2019). Tissue-specific regulation of Na^+^ and K^+^ transporters explains genotypic differences in salinity stress tolerance in rice. Front. Plant Sci..

[14] Wang X., Chen Z., Sui N. (2024). Sensitivity and responses of chloroplasts to salt stress in plants. Front. Plant Sci..

[15] Huang L., Li Z., Liu Q., Pu G., Zhang Y., Li J. (2019). Research on the adaptive mechanism of photosynthetic apparatus under salt stress: New directions to increase crop yield in saline soils. Ann. Appl. Biol..

[16] Khare T., Seth C.S., Kumar V. (2024). Sodium stress-induced oxidative damage and antioxidant responses during grain filling in Indica rice. Plant Cell Rep..

[17] Saleem M.A., Khan A., Tu J., Huang W., Liu Y., Feng N., Zheng D., Xue Y. (2025). Salinity stress in rice: Multilayered approaches for sustainable tolerance. Int. J. Mol. Sci..

[18] Ismail A.M., Horie T. (2017). Genomics, Physiology, and Molecular Breeding Approaches for Improving Salt Tolerance. Annu. Rev. Plant Biol..

[19] Bonilla P., Mackell D., Deal K., Gregorio G. (2002). RFLP and SSLP mapping of salinity tolerance genes in chromosome 1 of rice (*Oryza sativa* L.) using recombinant inbred lines. Philipp. Agric. Sci..

[20] Ren Z.H., Gao J.P., Li L.G., Cai X.L., Huang W., Chao D.Y., Zhu M.Z., Wang Z.Y., Luan S., Lin H.X. (2005). A rice quantitative trait locus for salt tolerance encodes a sodium transporter. Nat. Genet..

[21] Thomson M.J., de Ocampo M., Egdane J., Rahman M.A., Sajise A.G., Adorada D.L., Tumimbang-Raiz E., Blumwald E., Seraj Z.I., Singh R.K. (2010). Characterizing the *Saltol* quantitative trait locus for salinity tolerance in rice. Rice.

[22] Huyen L.T.N. (2013). Introgression the SALTOL QTL into Q5DB, the elite variety of Vietnam using marker-assisted-selection (MAS). Am. J. Biosci..

[23] Krishnamurthy S.L., Pundir P., Warraich A.S., Rathor S., Lokeshkumar B.M., Singh N.K., Sharma P.C. (2020). Introgressed *Saltol* QTL lines improves the salinity tolerance in rice at seedling stage. Front. Plant Sci..

[24] Tiwari K., Tiwari S., Kumar N., Sinha S., Krishnamurthy S.L., Singh R., Kalia S., Singh N.K., Rai V. (2024). QTLs and genes for salt stress tolerance: A journey from seed to seed continued. Plants.

[25] Suriyaarunroj D., Khongsuwan P., Lertna S., Hintung P., Budjun I., Boonmee N., Saleetoe S., Jumpagate R., Sripanom P., Siangliw J. (2018). RD73, a non-glutinous rice variety. Thai Rice Res. J..

[26] Vanavichit A., Kamolsukyeunyong W., Siangliw M., Siangliw J.L., Traprab S., Ruengphayak S., Chaichoompu E., Saensuk C., Phuvanartnarubal E., Toojinda T. (2018). Thai Hom Mali Rice: Origin and breeding for subsistence rainfed lowland rice system. Rice.

[27] Pamuta D. (2021). Development of Rice for Salt and Drought Tolerance Using Backcross Breeding and Marker Assisted Selection. Ph.D. Thesis.

[28] IRRI (2021). Phenotyping Protocols for Abiotic Stress Tolerance in Rice.

[29] Singh R.K., Kota S., Flowers T.J. (2021). Salt tolerance in rice: Seedling and reproductive stage QTL mapping come of age. Theor. Appl. Genet..

[30] Kanawapee N., Sanitchon J., Lontom W., Threerakulpisut P. (2012). Evaluation of salt tolerance at the seedling stage in rice genotypes by growth performance, ion accumulation, proline and chlorophyll content. Plant Soil.

[31] Prusty M.R., Kim S.R., Vinarao R., Entila F., Egdane J., Diaz M.G.Q., Jena K.K. (2018). Newly identified wild rice accessions conferring high salt tolerance might use a tissue tolerance mechanism in leaf. Front. Plant Sci..

[32] Nguyen T.T., Dwiyanti M.S., Sakaguchi S., Koide Y., Le D.V., Watanabe T., Kishima Y. (2022). Identification of a Saltol–independent salinity tolerance polymorphism in Rice Mekong Delta landraces and characterization of a promising line, Doc Phung. Rice.

[33] Moradi F., Ismail A.M. (2007). Responses of photosynthesis, chlorophyll fluorescence and ROS–scavenging systems to salt stress during seedling and reproductive stages in rice. Ann. Bot..

[34] Mohammadi R., Mendioro M.S., Diaz G.Q., Gregorio G.B., Singh R.K. (2014). Genetic analysis of salt tolerance at seedling and reproductive stages in rice (*Oryza sativa*). Plant Breed..

[35] Ahmadizadeh M., Vispo N.A., Calapit–Palao C.D., Pangaan I.D., Vina C.D., Singh R.K. (2016). Reproductive stage salinity tolerance in rice: A complex trait to phenotype. Indian J. Plant Physiol..

[36] Chapagain S., Pruthi R., Singh L., Subudhi P.K. (2024). Comparison of the genetic basis of salt tolerance at germination, seedling, and reproductive stages in an introgression line population of rice. Mol. Biol. Rep..

[37] Rodríguez Coca L.I., García González M.T., Gil Unday Z., Jiménez Hernández J., Rodríguez Jáuregui M.M., Fernández Cancio Y. (2023). Effects of sodium salinity on rice (*Oryza sativa* L.) cultivation: A review. Sustainability.

[38] Tariq F., Ma C., Zhao S. (2025). Integrative dynamics of cell wall architecture and plant growth under salt stress. Front. Plant Sci..

[39] Pamuta D., Siangliw M., Sanitchon J., Pengrat J., Siangliw J.L., Toojinda T., Theerakulpisut P. (2020). Photosynthetic performance in improved ‘KDML105’ rice (*Oryza sativa* L.) lines containing drought and salt tolerance genes under drought and salt stress. Pertanika J. Trop. Agric. Sci..

[40] Ma L., Li J., Li J., Huo Y., Yang Y., Jiang C., Guo Y. (2026). Plant salt–tolerance mechanisms: Classic signaling pathways, emerging frontiers, and future perspectives. Mol. Plant.

[41] Parvaiz A., Satyawati S. (2008). Salt stress and phyto–biochemical responses of plants—A review. Plant Soil Environ..

[42] Shao X., Gai D., Gao D., Geng Y., Guo L. (2021). Effects of salt–alkaline stress on carbohydrate metabolism in rice seedlings. Phyton–Inter. J. Exp. Bot..

[43] Thalmann M., Santelia D. (2017). Starch as a determinant of plant fitness under abiotic stress. New Phytol..

[44] Chen T., Niu Y., Yang C., Liang Y., Xu J. (2024). Screening of rice *(Oryza sativa* L.) genotypes for salinity tolerance and dissecting determinants of tolerance mechanism. Plants.

[45] Praphasanobol P., Chokwiwatkul R., Habila S., Chantawong Y., Buaboocha T., Comai L., Chadchawan S. (2025). Effects of salt stress at the booting stage of grain development on physiological responses, starch properties, and starch–related gene expression in rice (*Oryza sativa* L.). Plants.

[46] Li Y., Xue Y., Guan Z., Wang Z., Hou D., Zhao T., Lu X., Qi Y., Hao Y., Liu J. (2025). Salt stress responses of different rice varieties at panicle initiation: Agronomic traits, photosynthesis, and antioxidants. Plants.

[47] Su H., Wang W., Lu T., Hu W., Lin J., Fu W., Liang Y., Zeng Y., Fu G., Xiong J. (2025). Increased photosynthetic capacity and energy status contribute to higher grain yield in early rice. Int. J. Mol. Sci..

[48] Franzisky B.L., Geilfus C., Romo-Pérez M.L., Fehrle I., Erban A., Kopka J., Zörb C. (2021). Acclimatisation of guard cell metabolism to long–term salinity. Plant Cell Environ..

[49] Chaves M.M., Flexas J., Pinheiro C. (2009). Photosynthesis under drought and salt stress: Regulation mechanisms from whole plant to cell. Ann. Bot..

[50] Kobayashi N.I., Yamaji N., Yamamoto H., Okubo K., Ueno H., Costa A., Tanoi K., Matsumura H., Fujii-Kashino M., Horiuchi T. (2017). OsHKT1;5 mediates Na^+^ exclusion in the vasculature to protect leaf blades and reproductive tissues from salt toxicity in rice. Plant J..

[51] Zhang R., Wang Y., Hussain S., Yang S., Li R., Liu S., Chen Y., Wei H., Dai Q., Hou H. (2022). Study on the effect of salt stress on yield and grain quality among different rice varieties. Front. Plant Sci..

[52] Gerona M.E.B., Deocampo M.P., Egdane J.A., Ismail A.M., Dionisio-Sese M.L. (2019). Physiological responses of contrasting rice genotypes to salt stress at reproductive stage. Rice Sci..

[53] Rongsawat T., Pankasem N., Thianthavon T., Mangkalasane P., Aesomnuk W., Dumhai R., Ruengphayak S., Siangliw J.L., Siangliw M., Pitaloka M.K. (2025). QTL-seq identifies NAL1 and OsOFP19 as additive regulators of tiller number in rice (*Oryza sativa* L.). BMC Plant Biol..

[54] Zhang B., Wen Q., Feng W., Wang R., Liu W., Huang W., Khatab A., Li J., Xing Y. (2026). From green revolution to multigene revolution: Breeding high-yield rice by design. Mol. Plant.

[55] Solanki M., Yousuf F., Srivastava A., Vaikuntapu P.R., Molla K., Neeraja C.N., Thakur V., Barbadikar K.M., Sundaram R.M., Monhannath G. (2026). Powering Genome Editing in Rice by Harnessing Promising Gene Resources: A Comprehensive Roadmap. Physiol. Plant..

[56] Izquierdo J., Arriagada O., García–Pintos G., Ortiz R., García–Pintos M., García–Pintos M. (2024). On–farm foliar application of a humic biostimulant increases the yield of rice. Agron. J..

[57] Deng J., Liao X., Shi J., Xu Q., Yang X., Wang R., Ding J., Jia L., Jiang X., Yan S. (2026). Tiller–specific formulated fertilizer improves the population tiller quantity and yield of machine–transplanted rice. PLoS ONE.

[58] Zhang Y., Sultan H., Shah A., Mu Y., Li Y., Li L., Huang Z., Song S., Tao Y., Zhou Z. (2025). Regulation effect of seed priming on sowing rate of direct seeding of rice under salt stress. Front. Plant Sci..

[59] Yoshida S., Forno D.A., Cock J.H., Gomez K.A. (1976). Laboratory Manual for Physiological Studies of Rice.

[60] Lichtenthaler H.K. (1987). [34] Chlorophylls and carotenoids: Pigments of photosynthetic biomembranes. Methods in Enzymology.

[61] Barr H.D., Weatherley P.E. (1962). A re–examination of the relative turgidity technique for estimating water deficit in leaves. Aust. J. Biol. Sci..

[62] Ghoulam C., Foursy A., Fares K. (2002). Effects of salt stress on growth, inorganic ions and proline accumulation in relation to osmotic adjustment in five sugar beet cultivars. Environ. Exp. Bot..

[63] Patade V.Y., Bhargava S., Suprasanna P. (2011). Salt and drought tolerance of sugarcane under iso–osmotic salt and water stress: Growth, osmolytes accumulation, and antioxidant defense. J. Plant Interact..

[64] Numan Z.N., Ford C.J. (2015). Effect of ethyl alcohol on the dinitrosalicylic acid assay for reducing sugars. Res. Rev. J. Chem..

[65] Choudhury B., Mitra S., Biswas A.K. (2010). Regulation of sugar metabolism in rice (*Oryza sativa* L.) seedlings under arsenate toxicity and its improvement by phosphate. Physiol. Mol. Biol. Plants.

[66] Lou-Hing D., Zhang B., Price A.H., Meharg A.A. (2011). Effects of phosphate on arsenate and arsenite sensitivity in two rice (*Oryza sativa* L.) cultivars of different sensitivity. Environ. Exp. Bot..

[67] Bates L.S., Waldren R.P.A., Teare I.D. (1973). Rapid determination of free proline for water–stress studies. Plant Soil.

[68] Velikova V., Yordanov I., Edreva A. (2000). Oxidative stress and some antioxidant systems in acid rain–treated bean plants: Protective role of exogenous polyamines. Plant Sci..

[69] R Core Team (2023). R: A Language and Environment for Statistical Computing.

[70] Posit Team (2024). R Studio. Integrated Development Environment for R.

