# Supplementary material for: Salt Tolerance and Physiological Responses at the Seedling, Vegetative, and Reproductive Stages of Thai Jasmine Rice KDML105 and Its Genetically Improved Variety (RD73) and Line (TSKC1-144)
Source: Plants (Basel). 2026 Aug 28;15(17):2635. doi: 10.3390/plants15172635 (PMC13567463; doi:10.3390/plants15172635)
Supplement: Supplementary file 1 [file plants-15-02635-s001.zip › plants-4477942-supplementary.pdf]

Supplementary Tables for

Khampookhiaw et al. (2026)

Salt tolerance at the seedling, vegetative and reproductive stages of Thai jasmine rice KDML105 and its genetically improved variety (RD73) and line (TSKC1-144)

Table S1. Total chlorophyll, maximum quantum yield of PS II in the light (Fv'/Fm'), net photosynthesis rate (Pn), stomatal conductance (gs) and intercellular CO_2 concentration (Ci) of flag leaves of pot–grown rice plants under non–saline (Control) and salt stress (150 mM NaCl) conditions during the reproductive growth stages. Different lowercase letters within each growth stage indicated significant ($p \le 0.05$) difference among genotypes and between the control and stress condition. Different uppercase letters indicate significant ($p \le 0.05$) among growth stage on each genotype. The symbol (%) indicates the percent change in the stress condition relative to the control. Significant differences between the control and salt stress (($p \le 0.05$)) are denoted by *.

Growth stage	Variety/Line	Chlorophyll (mg g^{-1} FW)		%	Fv′/Fm′		%	Pn (μmol CO_2 m^{-2} s^{-1})		%	gs (mol H_2O m^{-2} s^{-1})		%	Ci		%
		**Control**	**Salt**		**Control**	**Salt**		**Control**	**Salt**		**Control**	**Salt**		**Control**	**Salt**	
**Booting**	KDML105	1.84 ab	1.80 ab	–2	0.615 a	0.519 a	–16	11.16 a	9.51 a	–15	0.146 ab	0.109 b	–26	215 a	251 a	17
	RD73	1.56 b	1.82 ab	17	0.490 a	0.473 a	–3	10.52 a	11.06 a	5	0.139 ab	0.201 a	45	232 a	220 a	–5
	TSKC1–144	2.01 a	1.74 ab	–13	0.505 a	0.476 a	–6	9.75 a	8.92 a	–9	0.129 b	0.123 b	–4	277 a	228 a	–18
	**Mean**	**1.80 A**	**1.79 A**	0	**0.537 A**	**0.489 A**	**–8**	**10.48 B**	**9.83 B**	**–6**	**0.138 C**	**0.144 A**	**5**	**241 A**	**232 A**	**–4**
**Flowering**	KDML105	1.71 a	1.68 a	–1	0.454 ab	0.481 ab	6	16.35 ab	9.94 c	–39*	0.190 a	0.164 a	–14	235 a	298 a	27
	RD73	1.72 a	1.78 a	4	0.500 ab	0.477 ab	–5	13.60 a–c	11.77 bc	–13	0.190 a	0.095 b	–50*	227 a	191 a	–16
	TSKC1–144	1.70 a	1.80 a	6	0.516 a	0.421 b	–18*	17.95 a	14.82 a–c	–17	0.193 a	0.167 a	–13	224 a	279 a	25
	**Mean**	**1.71 A–C**	**1.76 AB**	3	**0.490 A**	**0.460 AB**	**–6**	**15.97 A**	**12.18 A**	**–23**	**0.191 AB**	**0.142 A**	**–26**	**228 A**	**256 A**	**12**
**Milky**	KDML105	1.75 ab	1.72 ab	–2	0.447 a	0.426 a	–5	12.45 b	8.94 c	–28*	0.177 b	0.106 c	–40*	256 a	237 a	–7
	RD73	1.68 ab	1.56 b	–7	0.430 a	0.447 a	4	14.12 ab	9.51 c	–33*	0.187 b	0.083 c	–55*	255 a	194 b	–24*
	TSKC1–144	1.88 a	1.70 ab	–9	0.441 a	0.392 a	–11	15.83 a	9.47 c	–40*	0.236 a	0.108c	–54*	267 a	259 a	–3
	**Mean**	**1.77 AB**	**1.66 AB**	–6	**0.440 A**	**0.422 CD**	**–4**	**14.14 A**	**9.30 B**	**–34**	**0.200 AB**	**0.107 A**	**–46**	**259 A**	**230 A**	**–11**
**Dough**	KDML105	1.62 b	1.32 c	–19*	0.444 a	0.454 a	2	14.23 ab	7.52 c	–47*	0.169 bc	0.088 d	–48*	230 a	269 a	17
	RD73	1.48 bc	1.94 a	31*	0.505 a	0.428 a	–15	16.52 a	11.0 a–c	–33	0.231 ab	0.125 cd	–46*	277 a	231 a	–17
	TSKC1–144	1.60 bc	1.55 bc	–3	0.437 a	0.454 a	4	17.28 a	9.02 bc	–48*	0.287 a	0.128 cd	–55*	277 a	248 a	–10
	**Mean**	**1.57 C**	**1.60 B**	3	**0.463 A**	**0.446 BC**	**–3**	**16.01 A**	**9.20 B**	**–43**	**0.230 A**	**0.121 A**	**–46**	**261 A**	**249 A**	**–5**
**Mature**	KDML105	1.45 bc	1.53 ab	5	0.439 a	0.401 a	–19	12.72 a	4.53 c	–64*	0.135 a	0.048 b	–65*	234 a	253 a	8
	RD73	1.79 a	1.20 c	–33*	0.418 a	0.397 a	–5	10.81 ab	6.63 c	–39*	0.171 a	0.088 ab	–48	273 a	283 a	4
	TSKC1–144	1.59 ab	1.10 c	–31*	0.430 a	0.415 a	–4	8.49 bc	6.91 bc	–19	0.120 a	0.038 b	–69*	267 a	202 a	–24
	**Mean**	**1.61 BC**	**1.28 C**	–19	**0.476 A**	**0.405 D**	**–13**	**10.67 B**	**6.02 C**	**–41**	**0.153 BC**	**0.061 B**	**–61**	**257 A**	**246 A**	–4
***F*–test**		**2.65**	**9.89**		**3.25**	**6.62**		**10.05**	**8.18**		**3.94**	**6.71**		**0.90**	**0.47**	
***P*–value**		**0.042**	**0.000**		**0.018**	**0.0002**		**0.000**	**0.000**		**0.006**	**0.0002**		**0.469**	**0.759**	

F– and *P*–values for testing differences in the mean values across genotypes of each trait among rice growth stages under each salinity treatment.

Table S2. Relative water content (RWC) and electrolyte leakage (EL) of flag leaves of pot–grown rice plants under non–saline (Control) and salt stress (150 mM NaCl) conditions during the reproductive growth stages. Different lowercase letters within each growth stage indicated significant ($p \leq 0.05$) difference among genotypes and between the control and stress condition. Different uppercase letters indicate significant ($p \leq 0.05$) among growth stage on each genotype. The symbol (%) indicates the percent change in the stress condition relative to the control. Significant differences between the control and salt stress (($p \leq 0.05$)) are denoted by *

Growth stage	Variety/Line	RWC (%)		%	EL (%)		%
		Control	Salt		Control	Salt	
**Booting**	KDML105	92.55 a	93.06 a	1	16.8 bc	17.0 bc	1
	RD73	90.04 a	93.69 a	4	18.8 ab	21.5 a	15
	TSKC1–144	91.59 a	91.82 a	0	15.2 c	18.0 bc	18
	**Mean**	**91.39 B**	**92.86 A**	**2**	**16.9 C**	**18.8 BC**	**11**
**Flowering**	KDML105	91.44 a	91.18 a	0	22.5 a	26.6 a	18
	RD73	89.61 a	91.80 a	2	22.7 a	24.4 a	7
	TSKC1–144	91.18 a	92.46 a	1	22.1 a	24.7 a	12
	**Mean**	**90.75 B**	**91.81 A**	**1**	**22.5 A**	**25.3 A**	**12**
**Milky**	KDML105	93.04 a	94.00 a	1	21.2 a–c	22.5 a	6
	RD73	93.74 a	93.15 a	–1	21.3 ab	22.2 ab	4
	TSKC1–144	93.10 a	92.75 a	0	17.3 c	18.6bc	7
	**Mean**	**93.29 A**	**93.30 A**	**0**	**20.0 B**	**21.1 AB**	**6**
**Dough**	KDML105	91.50 ab	92.33 ab	1	11.3 a	12.5 a	10
	RD73	91.26 ab	90.76 b	–1	13.1 a	16.2 a	24
	TSKC1–144	93.35 a	91.33 ab	–2	10.2 a	14.4 a	41
	**Mean**	**92.04 AB**	**91.47 A**	**–1**	**11.5 D**	**14.4 C**	**25**
**Mature**	KDML105	91.03 a	87.78 a	–4	5.5 b	27.9 a	401*
	RD73	90.36 a	85.85 a	–5	5.2 b	22.7 a	331*
	TSKC1–144	91.10 a	88.39 a	–3	5.2 b	20.7 a	298*
	**Mean**	**90.83 B**	**87.34 B**	**–4**	**5.3 E**	**23.8 A**	**343**
***F*–test**		**4.31**	**11.42**		**69.85**	**6.37**	
***P*–value**		**0.004**	**0.000**		**0.000**	**0.0003**	

F– and *P*–values for testing differences in the mean values across genotypes of each trait among rice growth stages under each salinity treatment.

Table S3. Hydrogen peroxide (H_2O_2) and Thiobarbituric acid reactive substances (TBARS) and Proline in flag leaves of pot–grown rice plants under non–saline (Control) and salt stress (150 mM NaCl) conditions during the reproductive growth stages. Different lowercase letters within each growth stage indicated significant ($p \leq 0.05$) difference among genotypes and between the control and stress condition. Different uppercase letters indicate significant ($p \leq 0.05$) among growth stage on each genotype. The symbol (%) indicates the percent change in the stress condition relative to the control. Significant differences between the control and salt stress (($p \leq 0.05$)) are denoted by *.

**Growth stage**	**Variety/ Line**	**H_2O_2** (μmol g^{-1} DW)		%	**TBARS** (nmol g^{-1} DW)		%	**Proline** (μg g^{-1} DW)		%
		**Control**	**Salt**		**Control**	**Salt**		**Control**	**Salt**	
**Booting**	KDML105	0.72 a	0.73 a	0	0.31 ab	0.32 ab	5	613.4 a	760.6 a	24
	RD73	0.784 a	0.89 a	14	0.26 b	0.29 ab	15	532.2 a	613.0 a	15
	TSKC1–144	0.77 a	0.91 a	18	0.30 ab	0.36 a	18	597.5 a	689.1 a	15
	**Mean**	**0.76 A**	**0.85 AB**	**11**	**0.29 AB**	**0.32 A**	**12**	**581.07 A–C**	**687.9 AB**	**18**
**Flowering**	KDML105	0.50 b	0.73 ab	19	0.21 d	0.26 bc	19*	558.3 a	532.4 a	–5
	RD73	0.60 ab	0.60 ab	18	0.23 cd	0.27 ab	18*	535.3 a	549.6 a	3
	TSKC1–144	0.68 ab	0.78 a	27	0.23 cd	0.29 a	27*	542.2 a	603.3 a	11
	**Mean**	**0.60 B**	**0.71 B**	**22**	**0.23 C**	**0.28 B**	**22**	**545.3 C**	**561.8 B**	**3**
**Milky**	KDML105	0.58 b	0.85 ab	46	0.25 bc	0.27 bc	6	603.3 a	685.3 a	14
	RD73	0.87 ab	1.120 a	28	0.22 c	0.24 c	6	663.4 a	708.6 a	7
	TSKC1–144	0.65 b	0.76 ab	16	0.29 ab	0.32 a	13	652.6 a	671.0 a	3
	**Mean**	**0.71 AB**	**0.91 AB**	**29**	**0.26 B**	**0.28 B**	**9**	**639.8 A**	**688.3 AB**	**8**
**Dough**	KDML105	0.41 c	0.77 ab	86*	0.29 b	0.31 b	6	564.7 a	638.5 a	13
	RD73	0.60 a–c	0.81 a	35	0.27 b	0.29 b	7	556.5 a	623.8 a	12
	TSKC1–144	0.64 a–c	0.53 bc	–17	0.31 b	0.37 a	21*	615.8 a	621.5 a	1
	**Mean**	**0.56 B**	**0.71 B**	**28**	**0.30 A**	**0.33 A**	**12**	**579.0 BC**	**627.9 B**	**8**
**Mature**	KDML105	0.65 b	0.86 b	32	0.26 ab	0.30 ab	14	602.9 b	705.6 b	17
	RD73	0.83 b	0.88 b	5	0.26 ab	0.25 b	–5	597.9 b	631.0 b	6
	TSKC1–144	0.91 b	1.32 a	45*	0.29 ab	0.32 a	10	623.8 b	1004.9 a	61*
	**Mean**	**0.80 A**	**1.03 A**	**28**	**0.28 AB**	**0.30 AB**	**7**	**608.2 AB**	**780.5 A**	**28**
***F*–test**		3.72	2.95		6.4	2.92		2.85	3.27	
***P*–value**		0.009	0.028		0.001	0.029		0.032	0.017	

F– and *P*–values for testing differences in the mean values across genotypes of each trait among rice growth stages under each salinity treatment.

Table S4. Total sugar, non–reducing sugar (nRS), reducing sugar (RS), and starch content in flag leaves of pot–grown rice plants under non–saline (Control) and salt stress (150 mM NaCl) conditions during the reproductive growth stages. Different lowercase letters within each growth stage indicated significant ($p \leq 0.05$) difference among genotypes and between the control and stress condition. Different uppercase letters indicate significant ($p \leq 0.05$) among growth stage on each genotype. The symbol (%) indicates the percent change in the stress condition relative to the control. Significant differences between the control and salt stress (($p \leq 0.05$)) are denoted by *

Growth stage	Variety/Line	Total sugar (mg g^{-1} DW)		%	nRS (mg g^{-1} DW)		%	RS (mg g^{-1} DW)		%	Starch (mg g^{-1} DW)		%
		**Control**	**Salt**		**Control**	**Salt**		**Control**	**Salt**		**Control**	**Salt**	
**Booting**	KDML105	47.36 a	47.28 a	0	36.08 a	36.78 a	2	11.28 a	10.49 a	–7	10.51 ab	19.41 ab	85
	RD73	51.34 a	54.20 a	6	37.64 a	40.83 a	8	13.69 a	13.37 a	–2	14.01 ab	9.42 b	–33
	TSKC1–144	28.10 b	46.33 a	65*	17.29 b	35.40 a	105*	10.80 a	10.93 a	1	16.08 ab	24.33 a	51
	**Mean**	**42.27 C**	**49.28 D**	**17**	**30.34 C**	**37.67 D**	**24**	**11.93 A**	**11.60 C**	**–3**	**13.54 A**	**17.73 A**	**31**
**Flowering**	KDML105	29.40 b	46.07 a	57*	15.01 b	35.29 a	135*	14.38 ab	10.78 b	–25	7.22 ab	9.70 ab	34
	RD73	55.35 a	50.42 a	–9	41.09 a	38.24 a	–7	14.25 ab	12.17 ab	–15	5.76 b	8.46 ab	47
	TSKC1–144	31.85 b	32.50 b	2	14.44 b	17.84 b	24	17.40 a	14.65 ab	–16	10.71 a	8.09 ab	–24
	**Mean**	**38.87 C**	**43.00 D**	**11**	**23.52 C**	**30.46 D**	**30**	**15.35 A**	**12.54 BC**	**–18**	**7.90 B**	**8.75 B**	**11**
**Milky**	KDML105	67.52 ab	62.02 ab	–8	52.42 ab	44.79 b	–15	15.13 a	17.22 a	14	11.15 b	20.61 a	85*
	RD73	78.97 a	75.74 a	–4	64.80 a	61.33 ab	–5	14.16 a	14.40 a	2	15.79 ab	14.33 ab	–9
	TSKC1–144	53.60 b	65.16 ab	22	43.64 b	50.86 ab	17	9.96 b	14.30 a	43*	18.42 ab	15.48 ab	–16
	**Mean**	**66.70 B**	**67.64 C**	**1**	**53.63 B**	**52.33 C**	**–2**	**13.09 A**	**15.31 A**	**17**	**15.12 A**	**16.81 A**	**11**
**Dough**	KDML105	91.38 a	84.42 a	–8	78.03 a	71.04 a	–9	13.34 ab	13.37 ab	0	16.05	13.72	–15
	RD73	73.32 a	78.40 a	7	60.85 a	66.59 a	9	12.47 ab	11.80 b	–5	9.68	10.83	12
	TSKC1–144	81.85 a	75.65 a	–8	66.76 a	63.32 a	–5	15.08 a	12.32 b	–18*	14.71	12.70	–14
	**Mean**	**82.19 A**	**79.49 B**	**–3**	**68.55 A**	**66.99 B**	**–2**	**13.63 A**	**12.50 BC**	**–8**	**13.48 A**	**12.42 AB**	**–8**
**Mature**	KDML105	92.60 a	94.48 a	2	80.69 ab	79.61 ab	–1	11.91 a	14.87 a	25*	14.28 a	18.85 a	32
	RD73	89.15 a	94.00 a	5	76.50 b	79.43 ab	4	12.64 a	14.56 a	15	14.79 a	17.99 a	22
	TSKC1–144	91.16 a	112.70 a	24	76.36 b	100.59 a	32*	14.79 a	12.35 a	–17	17.98 a	10.97 a	–39
	**Mean**	**90.97 A**	**100.39 A**	**10**	**77.85 A**	**86.54 A**	**11**	**13.12 A**	**13.93 AB**	**6**	**15.69 A**	**15.94 A**	**2**
***F*–test**		27.31	34.73		30.28	32.32		2.26	3.77		3.97	3.53	
***P*–value**		0.000	0.000		0.000	0.000		0.074	0.008		0.006	0.012	

F– and *P*–values for testing differences in the mean values across genotypes of each trait among rice growth stages under each salinity treatment.

Table S5. Split–plot ANOVA showing levels of significant differences for total chlorophyll (chl.), net photosynthesis rate (Pn), starch content, total sugar, non–reducing sugar (nRS), reducing sugar (RS), relative water content (RWC), leaf and root Na^+, K^+ and Na^+/K^+, root length, plant height, leaf, stem, root and total fresh weight (FW) and dry weight (DW) of three rice genotypes KDML105, RD73, and TSCK1–144 grown under non–saline (control) and saline (150 mM NaCl) conditions at the vegetative growth stages.

Parameter	Salinity condition (S)					Rice varieties/line (G)					S x G				
	df	SS	MS	*F*–test	*P*–value	*df*	SS	MS	*F*–test	*P*–value	*df*	SS	MS	*F*–test	*P*–value
Total chl.	1	0.148	0.148	0.50	0.531	2	1.297	0.648	5.67	0.018	2	0.010	0.005	0.05	0.955
Pn–day 3	1	935.7	935.7	153	0.001	2	12.86	6.431	1.24	0.324	2	2.76	1.382	0.27	0.771
Pn–day 6	1	671.8	671.8	34.2	0.009	2	7.688	3.844	0.47	0.635	2	6.932	3.466	0.42	0.663
Pn–day 9	1	350.4	350.4	20.2	0.020	2	18.80	9.401	0.87	0.444	2	6.505	3.253	0.3	0.745
Starch	1	513.6	513.6	24.2	0.016	2	719.3	359.6	5.26	0.022	2	132.1	66.07	0.97	0.408
Total sugar	1	44.95	44.95	0.53	0.520	2	15.58	7.789	0.06	0.942	2	877.1	438.5	3.35	0.069
nRS	1	102.5	102.5	0.55	0.510	2	45.15	22.57	0.21	0.812	2	184.4	92.22	0.87	0.445
RS	1	11.19	11.19	0.63	0.484	2	11.77	5.889	0.65	0.538	2	55.06	27.53	3.05	0.084
RWC	1	210.0	210.0	36.8	0.009	2	17.33	8.667	1.69	0.226	2	32.33	16.16	3.15	0.077
EL	1	12.02	12.022	11.4	0.042	2	9.727	4.865	5.78	0.017	2	3.934	1.967	2.34	0.138
Leaf Na^+	1	15.92	15.92	455.6	0.000	2	0.495	0.247	15.64	0.000	2	0.364	0.182	11.51	0.001
Leaf K^+	1	0.369	0.369	2.13	0.240	2	1.119	0.559	3.12	0.081	2	1.747	0.873	4.87	0.028
Leaf Na^+/ K^+	1	2.620	2.620	122.1	0.001	2	0.321	0.160	4.60	0.032	2	0.293	0.147	4.21	0.041
Root Na^+	1	10.45	10.45	2009	0.000	2	0.086	0.043	2.58	0.117	2	0.090	0.045	2.69	0.108
Root K^+	1	0.614	0.614	10.08	0.050	2	0.228	0.114	3.43	0.066	2	0.039	0.019	0.59	0.571
Root Na^+/ K^+	1	24.23	24.23	503.2	0.000	2	0.963	0.481	32.17	0.000	2	0.562	0.281	18.77	0.000
Root length	1	723.8	723.8	216.4	0.001	2	0.726	0.363	0.1	0.905	2	25.56	12.78	3.54	0.061
Plant height	1	2350	2350	466.2	0.000	2	264.4	132.2	16.92	0.000	2	79.15	39.58	5.07	0.025
Shoot FW	1	187.6	187.6	133.3	0.001	2	55.94	27.97	21.38	0.000	2	1.523	0.762	0.58	0.573
Root FW	1	105.00	105.0	317.6	0.000	2	4.366	2.183	6.07	0.015	2	8.266	4.133	11.49	0.001
Total FW	1	579.10	579.1	192.6	0.001	2	82.85	41.42	19.63	0.000	2	14.58	7.291	3.45	0.065
Shoot DW	1	4.1660	4.166	36.76	0.009	2	0.790	0.395	1.75	0.215	2	0.930	0.465	2.06	0.170
Root DW	1	2.4066	2.406	127.4	0.001	2	0.023	0.011	0.22	0.809	2	0.013	0.00667	0.12	0.885
Total DW	1	12.470	12.47	166.5	0.001	2	0.615	0.307	0.65	0.538	2	0.7408	0.3704	0.78	0.478

Table S6. Split–plot ANOVA showing levels of significant differences for total chlorophyll (Chl), maximum quantum yield of PS II in the light (F_v'/F_m'), net photosynthesis rate (Pn), stomatal conductance (gs), relative water content (RWC), electrolyte leakage (EL), thiobarbituric acid reactive substances (TBARS), proline content, hydrogen peroxide (H_2O_2), total sugar, non–reducing sugar (nRS), reducing sugar (RS), starch content, leaf and root Na^+, K^+ and Na^+/K^+, plant height, dry biomass (leaf, stem, root and total dry weight), yield and yield component at the mature stage including panicle length, tiller, number of filled seeds panicle^{-1}, number of unfilled seeds panicle^{-1}, filled seed weight panicle^{-1}, unfilled seed weight panicle^{-1}, 100 seed weight, filled seed weight plant^{-1} and unfilled seed weight plant^{-1} of three rice varieties/line (KDML105, RD73, and TSCK1–144) grown under non–saline (control) and saline (150 mM NaCl) conditions during the reproductive growth stages.

Parameter	Salinity condition (S)					Rice varieties/line (G)					S x G				
	df	SS	MS	*F*–test	*P*–value	*df*	SS	MS	*F*–test	*P*–value	*df*	SS	MS	*F*–test	*P*–value
**Booting stage**															
Total Chl.	1	0.001	0.001	0.05	0.836	2	0.138	0.069	1.53	0.256	2	0.282	0.141	3.12	0.080
F_v'/F_m'	1	0.013	0.0136	0.56	0.508	2	0.035	0.017	1.54	0.254	2	0.007	0.003	0.32	0.734
Pn	1	2.509	2.509	1.11	0.368	2	8.860	4.433	0.74	0.497	2	4.853	2.426	0.41	0.675
gs	1	0.000	0.000	0.10	0.767	2	0.010	0.000	8.40	0.005	2	0.006	0.000	8.59	0.004
Ci	1	418.9	418.9	0.44	0.5547	2	3013.5	1506	0.70	0.5175	2	7320.6	3660.28	1.69	0.225
RWC	1	12.90	12.90	0.78	0.441	2	5.650	2.825	0.77	0.484	2	14.41	7.205	1.97	0.182
EL	1	21.67	21.67	6.95	0.077	2	61.01	30.50	6.61	0.011	2	8.969	4.484	0.97	0.406
TBARS	1	0.007	0.007	3.37	0.163	2	0.013	0.006	2.34	0.138	2	0.001	0.0008	0.28	0.763
Proline	1	68484	68483	1.65	0.289	2	52903	26451	1.09	0.367	2	5001	2500	0.10	0.903
H_2O_2	1	0.043	0.043	0.48	0.538	2	0.06696	0.033	0.67	0.530	2	0.021	0.010	0.21	0.811
Total sugar	1	294.3	294.3	4.24	0.131	2	996.45	498.2	4.59	0.033	2	386	193.4	1.78	0.210
nRS	1	322.4	322.43	7.37	0.072	2	735.1	367.5	2.97	0.089	2	354.4	177.2	1.43	0.277
RS	1	0.639	0.639	0.11	0.761	2	37.55	18.77	2.57	0.117	2	0.831	0.415	0.06	0.945
Starch	1	105.2	105.1	1.88	0.26	2	293.8	146.9	1.74	0.216	2	231.39	115.695	1.37	0.290
**Flowering stage**															
Total Chl.	1	0.013	0.013	7.90	0.067	2	0.015	0.007	0.36	0.703	2	0.016	0.008	0.39	0.685
F_v'/F_m'	1	0.005	0.005	1.65	0.288	2	0.002	0.001	0.54	0.594	2	0.015	0.007	3.76	0.054
Pn	1	86.21	86.21	2.68	0.200	2	64.90	32.47	4.40	0.036	2	22.26	11.13	1.51	0.260
gs	1	0.014	0.014	4.37	0.127	2	0.00669	0.003	1.80	0.206	2	0.0062	0.003	1.69	0.225
Ci	1	4633	4633	2.26	0.230	2	14323.7	7161	3.64	0.058	2	11993	5996	3.05	0.085
RWC	1	6.776	6.776	1.92	0.260	2	4.983	2.491	1.65	0.233	2	6.133	3.066	2.03	0.174
EL	1	47.27	47.26	17.1	0.025	2	6.488	3.243	0.17	0.849	2	5.926	2.963	0.15	0.861
TBARS	1	0.01442	0.0144	77.47	0.003	2	0.00283	0.0014	2.90	0.093	2	0.000	0.0002	0.59	0.568
Proline	1	1634	1633.95	0.19	0.688	2	4471	2235.3	0.42	0.665	2	7590	3795.0	0.71	0.509

Parameter	Salinity condition (S)					Rice varieties/line (G)					S x G				
	df	SS	MS	*F*–test	*P*–value	*df*	SS	MS	*F*–test	*P*–value	*df*	SS	MS	*F*–test	*P*–value
H_2O_2	1	0.0694	0.0694	35.37	0.009	2	0.07877	0.0393	0.85	0.450	2	0.05454	0.0272	0.59	0.569
Total sugar	1	102.46	102.46	1.29	0.339	2	1838.55	919.27	12.17	0.001	2	503.33	251.66	3.33	0.070
nRS	1	289.39	289.39	7.66	0.069	2	2253.81	1126.9	19.88	0.0002	2	573.01	286.5	5.05	0.025
RS	1	47.456	47.4564	2.69	0.199	2	53.728	26.864	4.16	0.042	2	2.345	1.1723	0.18	0.836
Starch	1	4.344	4.344	0.29	0.630	2	21.212	10.606	1.56	0.250	2	36.081	18.040	2.65	0.111
**Milky stage**															
Total Chl.	1	0.068	0.068	5.97	0.092	2	0.119	0.059	1.99	0.179	2	0.022	0.011	0.38	0.693
F_v'/F_m'	1	0.001	0.001	3.80	0.146	2	0.002	0.001	0.60	0.565	2	0.004	0.002	1.07	0.373
Pn	1	140.0	140.0	91.3	0.002	2	15.35	7.678	2.43	0.129	2	8.294	4.147	1.31	0.304
gs	1	0.051	0.051	16.3	0.027	2	0.011	0.005	13.36	0.001	2	0.001	0.000	1.63	0.237
Ci	1	5084.6	5084.6	9.12	0.056	2	5917.7	2958.9	4.85	0.028	2	3163.9	1582.0	2.59	0.116
RWC	1	0.000	0.000	0.01	0.944	2	1.685	0.842	0.50	0.617	2	2.780	1.390	0.83	0.460
EL	1	8.053	8.052	4.13	0.135	2	79.74	39.87	6.18	0.014	2	0.229	0.114	0.02	0.982
TBARS	1	0.0029	0.0029	6.15	0.089	2	0.022	0.011	12.49	0.001	2	0.0006	0.0003	0.38	0.692
Proline	1	14119	14119	1.56	0.299	2	7006	3503.0	0.33	0.727	2	4081	2040.6	0.19	0.829
H_2O_2	1	0.25222	0.2522	6.92	0.078	2	0.4370	0.21853	3.64	0.058	2	0.03083	0.01542	0.26	0.777
Total sugar	1	5.33	5.326	0.05	0.831	2	1360.2	680.108	4.85	0.028	2	343.37	171.685	1.23	0.327
nRS	1	10.04	10.040	0.09	0.781	2	1229.4	614.706	5.12	0.024	2	235.04	117.522	0.98	0.403
RS	1	29.677	29.676	18.75	0.022	2	65.574	32.7868	4.55	0.033	2	16.773	8.3867	1.16	0.345
Starch	1	17.069	17.068	1.10	0.372	2	14.395	7.1973	0.24	0.787	2	183.541	91.7704	3.11	0.081
**Dough stage**															
Total Chl.	1	0.007	0.007	0.91	0.411	2	0.220	0.110	2.36	0.136	2	0.610	0.305	6.54	0.012
F_v'/F_m'	1	0.001	0.001	0.85	0.423	2	0.002	0.001	0.38	0.692	2	0.011	0.005	2.07	0.169
Pn	1	278.2	278.2	13.1	0.036	2	37.73	18.86	3.46	0.065	2	7.985	3.993	0.73	0.501
gs	1	0.070	0.070	27.4	0.013	2	0.019	0.009	5.43	0.020	2	0.009	0.004	2.77	0.102
Ci	1	903.5	903.4	1.77	0.275	2	704.0	352.00	0.16	0.854	2	7961.0	3980	1.81	0.206
RWC	1	1.894	1.894	1.67	0.286	2	7.373	3.686	1.76	0.214	2	8.098	4.049	1.93	0.187
EL	1	48.69	48.69	1.14	0.363	2	34.97	17.48	1.49	0.264	2	9.522	4.761	0.41	0.675
TBARS	1	0.007	0.007	2.42	0.217	2	0.013	0.006	4.96	0.027	2	0.003	0.001	1.13	0.355
Proline	1	14361	14361	4.22	0.132	2	3285	1642	0.55	0.592	2	5647	2823.9	0.94	0.417
H_2O_2	1	0.140	0.140	3.76	0.147	2	0.080	0.040	1.32	0.304	2	0.2251	0.1125	3.67	0.057
Total sugar	1	43.56	43.56	0.18	0.702	2	631.9	315.9	1.10	0.364	2	181.80	90.89	0.32	0.734
nRS	1	14.68	14.67	0.06	0.818	2	557.21	278.6	0.96	0.410	2	172.68	86.33	0.30	0.747
RS	1	7.667	7.667	20.81	0.019	2	10.84	5.424	1.37	0.292	2	8.4566	4.228	1.06	0.375
Starch	1	6.747	6.747	0.18	0.698	2	92.35	46.17	2.58	0.116	2	14.812	7.406	0.41	0.669

Parameter	Salinity condition (S)					Rice varieties/line (G)					S x G				
	df	SS	MS	*F*–test	*P*–value	*df*	SS	MS	*F*–test	*P*–value	*df*	SS	MS	*F*–test	*P*–value
**Mature stage**															
Total Chl.	1	0.667	0.667	11.0	0.044	2	0.107	0.053	1.80	0.206	2	0.518	0.259	8.71	0.004
F_v'/F_m'	1	0.030	0.030	1.86	0.265	2	0.030	0.015	1.28	0.312	2	0.034	0.017	1.42	0.280
Φ PSII	1	0.002	0.002	4.22	0.132	2	0.010	0.005	0.95	0.414	2	0.017	0.008	1.53	0.255
ETR	1	689.3	689.2	4.22	0.132	2	2944	1472	0.94	0.416	2	4770	2385	1.53	0.256
Pn	1	129.9	129.9	17.8	0.024	2	5.069	2.535	0.71	0.511	2	44.20	22.10	6.18	0.014
gs	1	0.050	0.050	13.92	0.033	2	0.006	0.003	1.68	0.227	2	0.000	0.000	0.14	0.873
Ci	1	857.1	857.05	0.23	0.664	2	8586.9	4293.45	3.32	0.071	2	8492.4	4246.20	3.29	0.072
RWC	1	73.13	73.13	8.16	0.064	2	12.01	6.006	0.58	0.573	2	3.454	1.727	0.17	0.847
EL	1	2047	2047	49.2	0.005	2	62.40	31.20	0.45	0.649	2	50.20	25.10	0.36	0.704
TBARS	1	0.001	0.001	0.78	0.442	2	0.011	0.0000	3.97	0.047	2	0.002	0.001	1.03	0.385
Proline	1	178150	178150	54.19	0.005	2	179161	89581	5.03	0.026	2	135649	67825	3.81	0.052
H_2O_2	1	0.295	0.2950	12.25	0.039	2	0.545	0.272	5.35	0.021	2	0.132	0.0661	1.30	0.309
Total sugar	1	532.8	532.84	4.19	0.133	2	484.1	242.049	0.94	0.416	2	448.9	224.4	0.88	0.441
nRS	1	453.3	453.32	4.22	0.132	2	492.09	246.04	1.05	0.381	2	739.8	369.9	1.57	0.247
RS	1	3.965	3.965	0.23	0.666	2	0.215	0.1074	0.02	0.977	2	32.90	16.45	3.57	0.060
Starch	1	0.371	0.370	0.01	0.922	2	21.48	10.741	0.39	0.686	2	160.2	80.11	2.89	0.094
Leaf Na^+	1	9.55	9.550	1051	0.000	2	0.950	0.4760	22.61	0.000	2	0.704	0.352	16.7	0.0003
Leaf K^+	1	5.041	5.041	196.3	0.001	2	0.419	0.2096	7.16	0.009	2	0.124	0.062	2.12	0.162
Leaf Na^+/ K^+	1	1.285	1.285	4144	0.000	2	0.1910	0.0955	57.36	0.000	2	0.113	0.056	34.11	0.000
Root Na^+	1	2.982	2.982	457.6	0.0002	2	0.2553	0.1276	4.90	0.027	2	0.094	0.047	1.81	0.205
Root K^+	1	0.038	0.038	138.2	0.001	2	0.0117	0.0058	6.52	0.012	2	0.0002	0.0001	0.13	0.883
Root Na^+/ K^+	1	89.36	89.36	324.7	0.0004	2	5.225	2.612	2.64	0.111	2	2.097	1.0483	1.06	0.376
Plant height	1	160.1	160.1	2.06	0.246	2	92.90	46.44	0.80	0.471	2	47.90	23.94	0.41	0.670
Panicle length	1	3.7052	3.705	1.68	0.285	2	1.2139	0.6069	0.85	0.453	2	0.088	0.04402	0.06	0.9407
Tiller	1	1.042	1.041	0.48	0.536	2	78.250	39.125	24.50	0.0001	2	0.583	0.2917	0.18	0.8354
Number of filled seeds panicle^{-1}	1	2185.0	2185	36.53	0.009	2	5989.7	2994.8	16.10	0.0004	2	1.6	0.79	0.00	0.9958
Number of unfilled seeds panicle^{-1}	1	1751.04	1751	11.51	0.042	2	1085.58	542.79	3.62	0.0590	2	63.58	31.79	0.21	0.8121
Filled seed weight panicle^{-1}	1	3.4051	3.405	36.90	0.009	2	4.3474	2.1737	16.97	0.0003	2	0.1174	0.05872	0.46	0.6429
Unfilled seed weight panicle^{-1}	1	0.04682	0.0468	10.31	0.048	2	0.01701	0.0085	2.16	0.1582	2	0.00351	0.00175	0.45	0.6509
100 seed weight	1	0.15200	0.1520	22.56	0.017	2	0.16451	0.0822	37.44	0.0000	2	0.00386	0.00193	0.88	0.4407

Parameter	Salinity condition (S)					Rice varieties/line (G)					S x G				
	df	SS	MS	*F*–test	*P*–value	*df*	SS	MS	*F*–test	*P*–value	*df*	SS	MS	*F*–test	*P*–value
Filled seed weight plant^{-1}	1	364.65	364.65	130.5	0.001	2	594.48	297.24	7.32	0.0083	2	8.64	4.322	0.11	0.8999
Unfilled seed weight plant^{-1}	1	3.7604	3.7604	9.03	0.057	2	5.7065	2.85323	14.38	0.0007	2	0.7927	0.39633	2.00	0.1784
Leaf DW	1	63.25	63.24	3.95	0.141	2	725.6	362.8	20.9	0.000	2	8.500	4.251	0.25	0.786
Stem DW	1	5280.	5280	35.2	0.009	2	17591	8795	32.1	0.000	2	320.7	160.3	0.59	0.572
Root DW	1	0.001	0.002	0.00	0.994	2	509.3	254.6	8.08	0.006	2	4.489	2.245	0.07	0.931
Total DW	1	6506	6506	18.5	0.023	2	32924	16462	33.0	0.000	2	304.7	152.3	0.31	0.742
